# Exploration of phytoconstituents of Medhya Rasayana herbs to identify potential inhibitors for *cerebroside sulfotransferase* through high-throughput screening

**DOI:** 10.3389/fmolb.2024.1476482

**Published:** 2024-10-09

**Authors:** Nivedita Singh, Anil Kumar Singh

**Affiliations:** Department of Dravyaguna, Faculty of Ayurveda, Institute of Medical Sciences, Banaras Hindu University, Varanasi, Uttar Pradesh, India

**Keywords:** *cerebroside sulfotransferase*, substrate reduction therapy, metachromatic leukodystrophy, inhibition, drug, active site

## Abstract

*Cerebroside sulfotransferase* (CST) is a key enzyme in sulfatide biosynthesis and regulation of the myelin sheath in the nervous system. To counter sulfatide accumulation with the deficiency of aryl sulfatase A, CST is considered a target protein in substrate reduction therapy in metachromatic leukodystrophy. In this study, 461 phytoconstituents from four herbs of Medhya Rasayana were screened using multi-pronged virtual screening methods including molecular docking, molecular dynamics (MD) simulation, and reverse pharmacophore analysis. The initial screening of the top 15 hits was based on the binding affinity of the compounds toward the CST substrate-binding site using the lowest free energy of a binding score cutoff of ≤ −7.5 kcal/mol, with the number of conformations in the largest cluster more than 75. The absorption, distribution, metabolism, and excretion (ADME) and toxicity-based pharmacokinetic analysis delivered the top four hits: 18alpha-glycyrrhetinic acid, lupeol, alpha carotene, and beta-carotene, with high blood–brain barrier permeability and negligible toxicity. Furthermore, a 100-ns simulation of protein–ligand complexes with a trajectory analysis of structural deviation, compactness, intramolecular interactions, principal component analysis, free energy landscape, and dynamic cross-correlation analysis showed the binding potential and positioning of the four hits in the binding pocket. Thus, an in-depth analysis of protein–ligand interactions from pre- and post-molecular dynamics simulation, along with reverse pharmacophore mapping, suggests that 18alpha-glycyrrhetinic acid is the most potent and specific CST inhibitor, while beta-carotene could be considered the second most potent compound for CST inhibition as it also exhibited overall stability throughout the simulation. Therefore, the computational drug screening approach applied in this study may contribute to the development of oral drugs as a therapeutic option for metachromatic leukodystrophy.

## Graphical Abstract

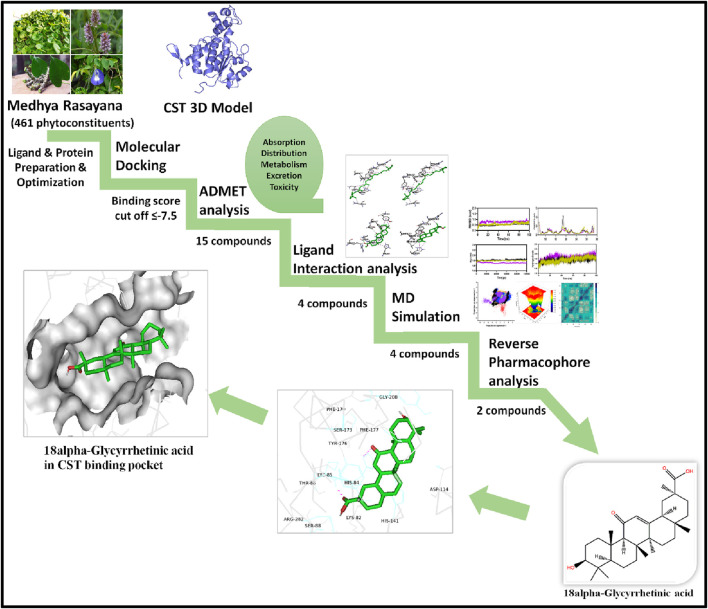

## 1 Introduction

Metachromatic leukodystrophy (MLD) is an autosomal recessive lysosomal storage neurodegenerative disorder, pathologically characterized by progressive motor and cognitive dysfunction (Miyake et al., 2021; Lamichhane and Cabrero, 2023; Penati et al., 2017; Singh and Singh, 2024b). MLD is caused by the deficiency of aryl sulfatase A (ARSA), which leads to the accumulation of sulfatide, the main glycolipid involved in stabilizing the myelin sheath equilibrium in the central and peripheral nervous systems (Sessa et al., 2016; Fumagalli et al., 2022). Sulfatide accumulation may cause various pathophysiological conditions, including progressive demyelination, neuroinflammation, communication gap, astrocyte dysfunction, developmental delay, speech disorder, and in severe cases it causes death within 5–6 years of the early onset of the disease (Miyake et al., 2021; Lamichhane and Cabrero, 2023; Fumagalli et al., 2022). Based on the age of onset, MLD is categorized into three major clinical types: late-infantile (≤30 months); juvenile, which is subdivided into early juvenile [30 months–6 years] and late juvenile [7–16 years]; and adult MLD (≥17 years) (Miyake et al., 2021; Fumagalli et al., 2022; Chang et al., 2024). Worldwide, the prevalence of MLD is 1.4–1.8 in 100,000 or 1 in 40,000, bringing it to the loop of rare diseases of greater concern (Lamichhane and Cabrero, 2023; Chang et al., 2024; Shaimardanova et al., 2020).

To date, more than 280 mutations have been reported in the *ARSA* gene, which poses a major challenge in the success of existing therapeutic options, including gene therapy, hematopoietic stem cell therapy, enzyme replacement therapy, and chaperone therapy, thus making these options costly and, therefore, demanding a case-by-case approach that leads the treatment of this rare disease beyond the reach of the larger population (Shaimardanova et al., 2020; Kurtzberg, 2022; Eichler et al., 2022; Fernández-Pereira et al., 2021; Sevin and Deiva, 2021). Another major obstacle to the success of existing therapies is the blood–brain barrier (BBB) in delivering the therapeutic gene or enzyme to the target site (Miyake et al., 2021). In contrast to existing therapies, which are mainly ARSA-dependent, a new approach of substrate reduction therapy (SRT) can be an alternate therapeutic option for metachromatic leukodystrophy. SRT has shown its success in various single-gene disorders including some key lysosomal storage disorders (Mistry et al., 2023; Komada et al., 2021; Istaiti et al., 2022). SRT focuses on the development of oral drugs to treat pathophysiological conditions. Miglustat and eliglustat are the two FDA-approved oral drugs for Gaucher’s disease (Mistry et al., 2023; Komada et al., 2021; Istaiti et al., 2022).

In MLD, the therapeutic focus in substrate reduction therapy is the development of a specific, potent, and competitive inhibitor which targets the catalytic action of the rate-limiting enzyme, *cerebroside sulfotransferase*, that is involved in the biosynthesis of sulfatides (Singh and Singh, 2024b; Yaghootfam et al., 2007; Li et al., 2020). To date, the development in substrate reduction therapy has been at the nascent stage in MLD. Experimental data with available CST inhibitors are limited, even though the full-length CST sequence and cDNA are available (Li et al., 2020). The lack of structural information of proteins due to the unavailability of the three-dimensional structure of CST has been a major roadblock in initiating preliminary *in silico*-based drug discovery, which has great potential in screening drug-like candidates in the shortest possible time with less expenses. The development of the three-dimensional homology model of CST by our group, which used a multipronged modeling and validation approach, is a breakthrough in SRT research in MLD (Singh and Singh, 2024a). This study uses this model for the screening of phytoconstituents from Medhya Rasayana.

Medhya Rasayana is a group of Ayurvedic medicinal herbs with neuroprotective effects against various neurodegenerative diseases and known to improve cognitive function and neural tissue regeneration, retard brain aging, and balance mental health (Kulkarni et al., 2012; Sarokte and Rao, 2013; Rashmi et al., 2017). Medhya Rasayana comprises four major herbs: Mandukaparni (*Centella asiatica* Linn.), Yastimadhu (*Glycyrrhiza glabra* Linn.), Guduchi (*Tinospora cordifolia* Miers), and Shankhpushpi (*Convolvulus pluricaulis* Chois) (Kulkarni et al., 2012). These herbs are rich in phytoconstituents with unique bioactive properties. This study focuses on identifying potent and specific bioactive compounds as inhibitors against CST through multipronged *in silico* studies. High-throughput virtual screening is the most common and reliable strategy used to identify the potent and specific inhibitors and potential lead molecules from the diverse dataset of small molecules at low cost and rapid pace using the recent advancement in the field of bioinformatics (Singh et al., 2018; Singh et al., 2017; Yasuo and Sekijima, 2019). With this perspective, the present study opens the door for many futuristic studies to achieve a marketed oral drug for metachromatic leukodystrophy that could bring this hereditary disease within the reach of a possible cost-effective treatment loop.

## 2 Methodology

### 2.1 Resources

The computational study was executed using the high-performance super-computing facility, PARAM Shivay, installed at the Indian Institute of Technology, Banaras Hindu University, Varanasi, India, with a capacity of 837 TFLOPS with Intel(R) Xeon(R) Gold 6148 CPU @2.40 GHz and 40 CPUs per node. This study used key software programs including AutoDock 4.2, GROMACS 2023, Origin 2024, Chimera 1.17.3, PyMOL, Discovery Studio visualizer, VMD, and Bio3D tool with R package and key webtools including PharmMapper, pkCSM, SwissADME, and ProTox 3.0.

### 2.2 Protein structural analysis and receptor grid generation

For screening of bioactive phytoconstituents, the 3D model of CST developed in our earlier study was used as a receptor. The model comprises 69–336 amino acid residues of the full-length protein with 423 residues and covers the entire catalytic region of the protein with a sulfuryl acceptor substrate (galactocerebroside)-binding site and a sulfuryl donor co-substrate (PAPS)-binding site on a linear horizontal plane (Singh and Singh, 2024a; Singh and Singh, 2024b). The model protein was processed using AutoDock 4.2 by the removal of water molecules and heteroatoms and the addition of hydrogen atoms with proper assignment of atom type and Gasteiger charge to generate a PDBQT file of the protein. A grid box of 90 × 90 × 90 Å and spacing of 0.253 Å around the protein active site was generated considering the key residues LYS82, HIS84, LYS85, HIS141, PHE170, TYR176, PHE177, TYR203, and ARG202, and a grid file (.gpf) was created.

### 2.3 Ligand preparation

We used a set of compounds from the Indian Medicinal Plants, Phytochemistry, And Therapeutics (IMPPAT 2.0) online database (Vivek-Ananth et al., 2023; Mohanraj et al., 2018). The 3D (.mol2) structures of 81 compounds from *C. asiatica* Linn (Mandukaparni), 310 from *G. glabra* Linn (Yastimadhu), 52 from *T. cordifolia* Miers (Guduchi), and 18 from *C. pleuricaulis* Chois (Shankhpushpi) were converted to .pdbqt files after energy minimization and assigning proper atom types using AutoDock Raccoon, a virtual screening file preparation tool (Forli et al., 2016).

Following the preparation of the protein, ligands, and grid files, AutoDock Raccoon was further used for the preparation of docking (.dpf) files for each ligand and the subsequent arrangement of all files in a single separate folder for each protein–ligand complex with the generation of a single virtual screening script file (.sh) for performing molecular docking-based virtual screening of all ligands simultaneously under the Linux environment. The parameters used for the generation of .dlg files for docking run were 100 GA run, a population size of 300, maximum number of generations of 27,000, and maximum number of evaluations of 25,000,000. AutoDock applied the Lamarckian genetic algorithm and a gradient-based local search method for protein–ligand interactions. The pKi and ligand efficiency are two major parameters for assessing the binding potential of ligands in the active site. The best-docked conformation was selected, processed using custom Python scripts, and visualized using .pdb visualization tools including PyMOL and Discovery Studio Visualizer to analyze protein–ligand interactions and ligand-binding patterns in the active site.

### 2.4 *In silico* drug-likeness properties, ADME, and toxicity analysis

The selected top hits based on the binding score and number of conformations in the largest cluster were subjected to pharmacokinetic property analysis using pkCSM, SwissADME, and ProTox webservers (Banerjee et al., 2018; Daina et al., 2017; Pires et al., 2015). The canonical Simplified Molecular Input Line Entry System (SMILE) of the selected compounds was used as the entry data in these servers for their pharmacokinetic and drug-likeness property analysis through adsorption, distribution, metabolism, and excretion along with toxicity studies.

### 2.5 Molecular dynamics simulations

All atom molecular dynamic (MD) simulations were performed using the GROMACS 2023.1 software package using the CHARMM27 all-atom additive force field. For the simulation, a dodecahedron simulation box was created with a minimum distance of 1.2 Å from the box edge, and periodic boundary conditions were applied to minimize the edge effect. Each box was solvated with the TIP3P water solvation model, and the charges on the system were then neutralized by the addition of chloride (Cl^−^) ions, and thereafter, the solvated system was energy-minimized using the steepest descent algorithm. The energy-minimized system was then equilibrated to 1,000-ps (1 ns) NVT simulation with a time step of 2 fs at 300 K. Next, the system was equilibrated to 1,000-ps NPT simulations with a time step of 2.0 fs at 300 K. The LINCS algorithm was used to constrain the bond lengths. The final MD simulation run was performed for 100 ns, and trajectories were analyzed using different MD parameters including root mean square deviation (RMSD), root mean square fluctuation (RMSF), radius of gyration (*R*g), solvent-accessible surface area (SASA), hydrogen bonds, principal component analysis (PCA), and dynamic cross-correlation matrix (DCCM) analysis of the protein–ligand complexes.

### 2.6 Cross-target prediction

The PharmMapper server was used to identify a wide range of targets using an innovative reverse pharmacophore mapping approach (Wang et al., 2017; Liu et al., 2010). The best mapping poses of the submitted molecule (.mol2) were aligned against all target proteins available in PharmTargetDB. The algorithm used to perform the reverse pharmacophore matching protocol comprises a sequential combination of triangle hashing (TriHash) and genetic algorithm (GA) optimization (Liu et al., 2010). Based on the calculated highest fit-score (cutoff >5.0) between the small compound and the pharmacophore models, the probable protein targets were ranked. It was imperative to check the disease-causing potential of the targets.

## 3 Results

### 3.1 Molecular docking-based virtual screening

Virtual screening is a computational approach used to screen libraries of compounds available in databases against the target protein to identify a potential drug candidate for a targeted disease (Singh et al., 2018; Singh et al., 2021; Zare et al., 2024; Pirolli et al., 2023). In the CST-led research to develop substrate reduction therapy for metachromatic leukodystrophy, the state-of-the-art *in silico* approach could be the most promising and directional method for future studies. Toward this approach, the development of a homology model of the CST protein using various computational algorithms is an important step (Singh and Singh, 2024a). In the present study, this 3D model was utilized for screening specific and potent phytoconstituents of four major herbs of Medhya Rasayana. Out of a total of 461 bioactive compounds, 81 compounds from *C. asiatica* Linn., 310 from *G. glabra* Linn., 52 from *T. cordifolia* Miers, and 18 from *C. pleuricaulis* Chois were screened against CST, which are detailed in Supplementary Tables S1–S4. In the initial level of screening, using the lowest free energy of a binding cutoff of ≤ −7.5 kcal/mol and the number of conformation cutoff of ≥75 in the largest conformation cluster, the top 15 compounds were selected, i.e., 3 from *C. asiatica* and 12 from *G. glabra*. Compounds belonging to *T. cordifolia and P. Chois* were found to be less potent and less specific toward CST and, hence, were not considered for further study. The docking score of these top 15 compounds falls between −7.57 and −10.32 kcal/mol. With a ligand efficiency of ≤0.19 per non-hydrogen atom, the top 15 compounds were considered promising for drug-likeness property analysis. Apart from α- and β-carotene, all 13 compounds strictly followed the Lipinski rule of 5 parameters in terms of molecular mass (<500), number of hydrogen bond donors (<5), hydrogen bond acceptors (<10), number of rotatable bonds, and topological surface area (TPSA). Despite failing at the Lipinski rule of five parameters, α- and β-carotene were considered for further studies because of their wider therapeutic potential in neurodegenerative diseases, and these molecules have already been tested in the human body (Banerjee et al., 2023; Abrego-Guandique et al., 2023; Hira et al., 2019; Narisawa et al., 1996). Additionally, these compounds showed good lipophilicity with a log *p*-value > 0, which is imperative for compounds to cross the lipophilic membrane and, thus, strengthen the movement of these molecules to the target site. Therefore, all 15 hits were considered for absorption, distribution, metabolism, and excretion (ADME) and toxicity analysis to eliminate false positives from this list, which is provided in Table 1.

**TABLE 1:** Binding score and physiochemical properties of the top 15 compounds.

Sl. no.	Compounds	Common name	Source	Binding affinity (kcal/mol)	Ligand efficiency (kcal/mol/non-h-atom)	pKi (nM)	Molecular weight	Hydrogen bond donor	Hydrogen bond acceptor	Rotational bond	Ring	LogP	TPSA ( ${Å}^{2}$ )
1	IMPHY012226	18alpha-Glycyrrhetinic acid	*Glycyrrhiza glabra*	−10.32	−0.30	7.56	470.69	2	3	1	5	6.41	74.6
2	IMPHY002304	Liquoric acid	*Glycyrrhiza glabra*	−10.22	−0.29	7.49	484.68	2	4	1	6	5.4	83.83
3	IMPHY011842	11-Deoxoglycyrrhetinic acid	*Glycyrrhiza glabra*	−9.92	−0.30	7.27	456.71	2	2	1	5	7.23	57.53
4	IMPHY015702	Glabric acid	*Glycyrrhiza glabra*	−9.67	−0.28	7.09	486.69	3	4	1	5	5.38	94.83
5	IMPHY011880	Ursolic acid	*Centella asiatica*	−9.28	−0.28	6.80	456.71	2	2	1	5	7.09	57.53
6	IMPHY015709	28-Hydroxyglycyrrhetinic acid	*Glycyrrhiza glabra*	−9.21	−0.26	6.75	486.69	3	4	2	5	5.39	94.83
7	IMPHY010136	18α-Hydroxyglycyrrhetinic acid	*Glycyrrhiza glabra*	−9.03	−0.26	6.61	486.69	3	4	1	5	5.53	94.83
8	IMPHY005115	Ursolic acid lactone	*Centella asiatica*	−8.54	−0.26	6.26	456.71	1	3	0	6	6.76	46.53
9	IMPHY001586	Isoglabrolide	*Glycyrrhiza glabra*	−8.44	−0.25	6.18	468.68	1	4	0	6	6.01	63.6
10	IMPHY012473	Lupeol	*Glycyrrhiza glabra*	−8.35	−0.27	6.12	426.73	1	1	1	5	8.02	20.23
11	IMPHY006740	Glabrolide	*Glycyrrhiza glabra*	−8.23	−0.24	6.04	468.68	1	4	0	6	5.86	63.6
12	IMPHY011609	alpha-Carotene	*Centella asiatica*	−7.90	−0.20	5.79	536.89	0	0	10	2	12.46	0
13	IMPHY011707	beta-Carotene	*Glycyrrhiza glabra*	−7.64	−0.19	5.59	536.89	0	0	10	2	12.61	0
14	IMPHY011620	Lutein	*Glycyrrhiza glabra*	−7.59	−0.19	5.57	568.886	2	2	10	2	10.4	40.46
15	IMPHY001500	Hispaglabridin B	*Glycyrrhiza glabra*	−7.57	−0.19	5.55	390.479	1	4	1	5	5.48	47.92

### 3.2 Absorption, distribution, metabolism, excretion, and toxicity profiling

**FIGURE 1:**
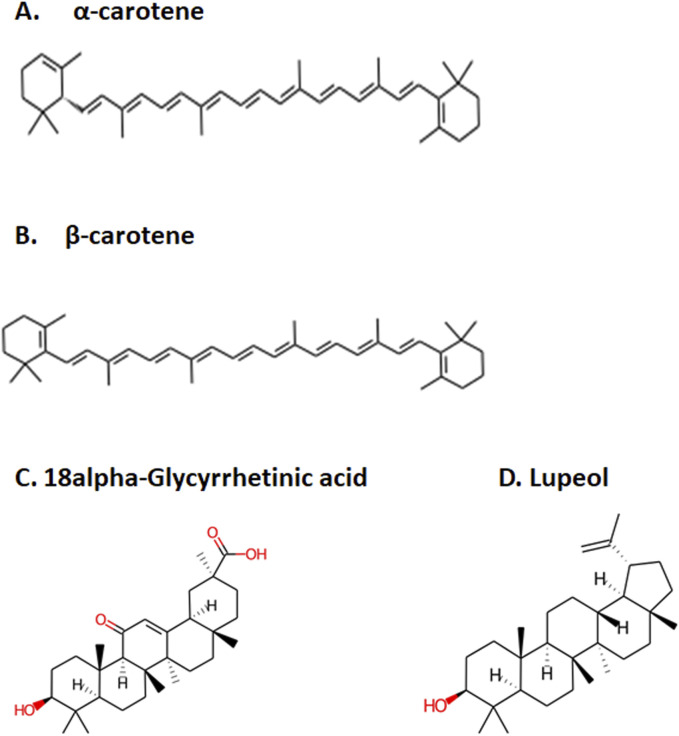
Top four selected compounds. **(A)** α-carotene. **(B)** β-carotene. **(C)** 18alpha-glycyrrhetinic acid. **(D)** lupeol.

After the administration of the drug in the body, it goes through absorption, distribution, metabolism, and excretion processes. In this process, the drug interacts with desirable or undesirable targets and causes a pharmacological impact (Zhang and Tang, 2018). Thus, the bioavailability of a drug depends on the rate of absorption, metabolism, and transportation of the drug to the target site (Zhang and Tang, 2018; Stielow et al., 2023). Human intestinal absorption and lipophilicity of compounds are other important parameters to be considered to ensure the easy diffusion of compounds. The cytochrome p450-based drug response to body metabolism exhibits genetic variability and plays a critical role in the detoxification of drugs and homeostasis (Zhao et al., 2021). In this study, CYP2C9, CYP2D6, and CYP3A4 inhibitors were used to analyze the drug response at the metabolism stage. Renal OCT2 is a renal transporter that plays an important role in determining the renal clearance or deposition of drugs (Zou et al., 2021; Wright, 2019). Except few, most of the selected compounds showed good intestinal absorption capacity, along with body metabolism and renal excretion potential. Since MLD is a brain disorder, the major deciding parameters for the potential drug candidates were BBB permeability, which screened four compounds—IMPHY012226 (18alpha-glycyrrhetinic acid), IMPHY012473 (lupeol), IMPHY011609 (alpha-carotene), and IMPHY011707 (beta-carotene)—with considerable blood–brain barrier permeability. In order to select a potential lead molecule, *in silico* toxicity study becomes another crucial criterion due to its accuracy, accessibility, and rapidity in preclinical-level screening and providing a potential lead scaffold for further optimization (Raies and Bajic, 2016; Hemmerich and Ecker, 2020; Yang et al., 2018). pkCSM and ProTox 3.0 are freely available *in silico* toxicity study servers that use the SMILE of each compound to evaluate various toxicity parameters including Ames mutagenicity, hepatotoxicity, and cytotoxicity (Banerjee et al., 2018; Pires et al., 2015). These four phytoconstituents belonged to class “4” of the ProTox-predicted toxicity class based on their LD_50_ range between 560 and 2,000 mg/kg, suggesting that the selected four compounds are mainly nontoxic. Table 2 provides the details of ADME and toxicity analysis of the top 15 hits, and Figure 1 shows the structural details of the best 4 compounds screened through ADME and toxicity analysis.

**TABLE 2:** Pharmacological profile of the top 15 ligand molecules that were derived from pkCSM, SwissADME, and ProTox webservers.

Sl. no.	Compounds common name	Absorption	Distribution	Metabolism			Excretion	Toxicity		
		Gastrointestinal absorption	BBB permeation	CYP2C9 inhibitor	CYP2D6 inhibitor	CYP3A4 inhibitor	Renal OCT2 substrate	Ames test	Hepatotoxicity	Predicted toxicity class 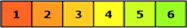
1	18alpha-Glycyrrhetinic acid	High	Moderate	No	No	No	No	No	No	4
2	Liquoric acid	High	Poor	No	No	No	No	No	No	3
3	11-Deoxoglycyrrhetinic acid	Low	Poor	No	No	No	No	No	No	4
4	Glabric acid	High	Poor	No	No	No	No	No	No	4
5	Ursolic acid	Low	Poor	No	No	No	No	No	Yes	4
6	28-Hydroxyglycyrrhetinic acid	High	Poor	No	No	No	No	No	No	4
7	18α-Hydroxyglycyrrhetinic acid	High	Poor	No	No	No	No	No	No	6
8	Ursolic acid lactone	High	Moderate	No	No	No	No	No	Yes	5
9	Isoglabrolide	High	Moderate	No	No	Yes	No	No	No	4
10	Lupeol	Low	High	No	No	No	No	No	No	4
11	Glabrolide	High	Poor	No	No	Yes	No	No	No	5
12	alpha-Carotene	High	High	No	No	No	No	No	No	4
13	beta-Carotene	High	High	No	No	No	No	No	No	4
14	Lutein	Low	Poor	No	No	No	No	No	No	2
15	Hispaglabridin B	High	Poor	Yes	No	Yes	Yes	No	No	4

### 3.3 Pre-MD/docking-based protein-ligand interaction pose analysis

**FIGURE 2:**
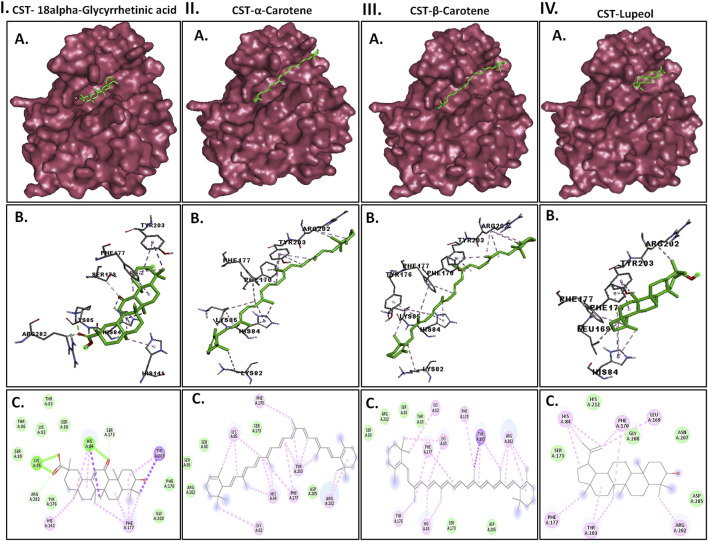
Interaction of the top four docked complexes, including **(A)** surface view (upper panel), **(B)** 3D view (middle panel), and **(C)** 2D view (lower panel) of the protein–ligand interaction at the substrate-binding site of CST protein in (I) CST–18alpha-glycyrrhetinic acid, (II) CST–α-carotene, (III) CST–β-carotene, and (IV) CST–lupeol complexes. In the 2D view, conventional hydrogen bond, pi–sigma, pi–alkyl, and van der Waals interactions are depicted in green, violet, pink, and light green, respectively.

Protein–ligand interaction analysis was imperative to obtain an insight into the binding pattern of compounds in the substrate-binding site of the CST protein. The substrate-binding site comprises a left-end polar site dominated by Lys85, Phe170, Lys82, and Ser88; a middle part flanked on both sides by His84 and His141; and an aromatic site at the right end dominated by Tyr203 and Phe170. With the lowest free binding energy of −10.32 kcal/mol and 98 similar conformations in the largest cluster, 18alpha-glycyrrhetinic acid was found to be the most potential ligand with the highest binding affinity among the 4 in the binding pocket of the CST protein. The compound consists of five steroidal rings with a carboxylic group attached at one end and a hydroxyl group at the other end. These ends determined the orientation of the compound toward the polar site based on the degree of polarity of the carboxylic group, which interacted with Lys85 by hydrogen bonding. One oxygen atom was attached by a double bond at the third ring in the middle of the compound positioned at the middle of the active site and was flanked on both sides by His84 and His141 and formed a hydrogen bond with His84. At the right side of the binding pocket, methyl groups located on the rightmost ring of the compound interacted with the residues Tyr203 and Phe177 via pi–sigma and pi–alkyl bonds, respectively (Figure 2I). Both α-carotene and β-carotene complexes shared similarity in the interaction pattern in the CST-binding pocket. α-Carotene and β-carotene are isomers, differing in the position of the double bond on the ring at one end oriented toward the polar site in the binding pocket. Lys82 and Lys85 played a critical role in the interaction with the isomeric fragment of these compounds. In α-carotene, the methyl group at the C2 position was oriented toward Lys85 and, thus, interacted with it, whereas in β-carotene, the methyl group at the C2 position was oppositely oriented and away from Lys85. In the CST–β-carotene complex, the two methyl groups at the C6 position were oriented toward Lys85 and interacted via the pi–alkyl interaction, whereas in the CST–α-carotene complex, the methyl groups at the C6 carbon were positioned away from Lys85 and interacted with Lys82. The aliphatic chain between the two aromatic rings interacted in a nearly similar fashion in both complexes with key residues Phe177, Tyr203, phe170, and His84, while Arg202 interacted with the aromatic ring on the right side of the binding pocket (Figures 2II, III). In contrast to the other three compounds that broadly occupied the active site core, in the CST–lupeol complex, the ligand predominantly occupied the right side of the binding pocket, which might be due to its small size and the missing of a polar carboxylic group at one end as it was in 18alpha-glycyrrhetinic acid. The lack of occupancy of the polar site by lupeol is due to its five-carbon nonpolar ring that interacted strongly at the aromatic site of the binding pocket (Figure 2IV), while the six-membered polar ring in 18alpha-glycyrrhetinic acid stretched the compound toward the polar site and, thus, was strongly accommodated in the active site (Figure 2I). The details of the interaction types are given in Table 3. To further understand the protein–ligand interaction under a dynamic environment, all four compounds were considered for molecular dynamic simulations.

**TABLE 3:** Non-bonded interaction in docked protein–ligand conformation.

Sl. no.	Compound	Residue in contact	Bond type	Distance (Å)
1	18alpha-Glycyrrhetinic acid	Lys85	Conventional hydrogen bond	1.60
		Lys85	Conventional hydrogen bond	2.10
		His84	Conventional hydrogen bond	2.20
		His84	Pi–sigma	4.25
		His84	Pi–alkyl	4.24
		His84	Pi–alkyl	5.30
		His141	Pi–alkyl	4.61
		Phe177	Pi–alkyl	5.37
		Phe177	Pi–alkyl	6.35
		Phe177	Pi–alkyl	6.63
		Phe177	Pi–alkyl	6.58
		Tyr203	Pi–sigma	4.60
		Tyr203	Pi–alkyl	5.10
		Ser173	van der Waals	3.48
		Arg282	van der Waals	4.33
2	α-Carotene	Lys82	Pi–alkyl	5.08
		Lys85	Pi–alkyl	3.12
		Lys85	Pi–alkyl	4.29
		Lys85	Pi–alkyl	6.93
		His84	Pi–alkyl	4.85
		His84	Pi–alkyl	4.96
		His84	Pi-Alkyl	5.76
		Phe170	Pi–alkyl	5.01
		Phe177	Pi–alkyl	5.36
		Phe177	Pi–alkyl	5.53
		Arg202	Pi–alkyl	3.37
		Arg202	Pi–alkyl	3.96
		Tyr203	Pi–alkyl	3.12
		Tyr203	Pi–alkyl	3.75
		Tyr203	Pi–alkyl	5.04
		Tyr203	Pi–alkyl	5.54
3	β-Carotene	Lys82	Pi–alkyl	4.51
		Lys85	Pi–alkyl	4.07
		Lys85	Pi–alkyl	5.63
		His84	Pi–alkyl	3.83
		His84	Pi–alkyl	4.68
		Phe170	Pi–alkyl	5.18
		Tyr176	Pi–alkyl	5.05
		Phe177	Pi–alkyl	5.87
		Phe177	Pi–alkyl	6.24
		Arg202	Pi–alkyl	3.74
		Arg202	Pi–alkyl	4.38
		Arg202	Pi–alkyl	4.51
		Arg202	Pi–alkyl	5.83
		Tyr203	Pi–sigma	3.86
		Tyr203	Pi–alkyl	4.21
		Tyr203	Pi–alkyl	4.38
4	Lupeol	His84	Pi–alkyl	5.40
		His84	Pi–alkyl	5.84
		His84	Pi–alkyl	5.85
		Leu169	Pi–alkyl	5.32
		Phe170	Pi–alkyl	3.74
		Phe170	Pi–alkyl	5.50
		Phe177	Pi–alkyl	6.31
		Tyr203	Pi–alkyl	3.82
		Tyr203	Pi–alkyl	4.29
		Arg202	Pi–alkyl	4.18

### 3.4 Molecular dynamic simulation

MD simulation is a computational approach used to analyze and optimize the overall stability of the protein–ligand complexes under atomistic simulation conditions with a dynamic aqueous environment (Guterres and Im, 2020; Kurniawan and Ishida, 2022; Khan et al., 2016). MD simulation provides a cumulative idea about the movement of every atom or atom in the protein over the simulation time span to study important biological processes, including the impact of ligand binding on the overall protein dynamics and the way the macromolecule responds at the atomic level with the binding or unbinding of the ligand (Alrouji et al., 2023; Hollingsworth and Dror, 2018). In this study, to understand the in-depth dynamic behavior of the protein–ligand interaction, MD simulation was carried out for a time span of 100 ns to evaluate the strength and stability of the protein–ligand complexes through trajectory analysis with various parameters including RMSD, RMSF, Rg, SASA, and hydrogen bonding. We also carried out principal component analysis, free energy landscape analysis, and dynamic cross-correlation analysis to understand the dominant motions responsible for the binding pattern and stability of the protein–ligand complex.

#### 3.4.1 Structural deviation and flexibility with RMSD and RMSF analysis

**FIGURE 3:**
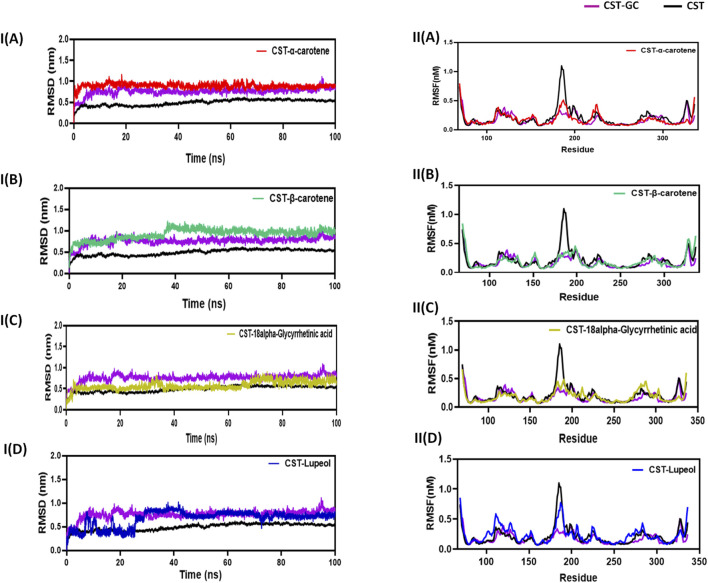
Structural dynamics of CST in complex with **(A)** α-carotene, **(B)** β-carotene, **(C)** 18alpha-glycyrrhetinic acid, and **(D)** lupeol. (I) Root mean square deviation (RMSD) plot over time, highlighting any deviations in the spatial structure of CST over a time scale of 100 ns from its original conformation; the plot used a protein backbone for considering deviation throughout simulation. (II) Root mean square fluctuation (RMSF) of residues in CST under different ligand-bound states during a time scale of 100 ns.

The RMSD measures the conformational deviation of the protein structure from the initial docked conformation to the final conformation under the dynamic aqueous environment over the simulation time span to ensure the stability of the predicted protein–ligand complex after ligand binding (Zare et al., 2024; Aier et al., 2016). In this study, in a simulation time of 100 ns, the CST–18alpha-glycyrrhetinic acid and CST–α-carotene complexes were found to be the most stable with negligible fluctuation, while the CST–β-carotene complex showed overall stability with little fluctuation at 40 ns The CST–lupeol complex showed initial rapid fluctuation, but after 25 ns, it also achieved stability. The average deviation found for CST, CST-GC, CST–18alpha-glycyrrhetinic acid, CST–α-carotene, CST–β-carotene, and CST-Lupeol was 0.49, 0.76, 0.57, 0.89, 0.92, and 0.67, respectively. Thus, RMSD simulation showed that the four complexes of CST could maintain overall structural stability during the simulation and no significant conformational changes occurred in the protein structure after ligand binding as the RMSD was maintained throughout the simulation either between the RMSD of free CST and the CST-GC complex or closer to the RMSD of the CST–GC complex, which is a good indication toward achieving competitive inhibition (Figures 3IA–D) (Singh and Singh, 2024a).

Following the protein conformational deviation studies with RMSD, protein structural fluctuation at the residue level was analyzed with RMSF calculation of the protein–ligand complex (Martínez, 2015; Paul et al., 2022). In this study, the fluctuations in the residues of the CST protein in the presence of the selected ligand were compared with the RMSF of the free CST and CST-GC complex. The average RMSF of free CST, CST-GC, CST–18alpha-glycyrrhetinic acid, CST–α-carotene, CST–β-carotene, and CST–lupeol was 0.15, 0.17, 0.18, 0.176, 0.18, and 0.23, respectively. As depicted in (Fig 3IIA–D), a wider residual fluctuation was clearly observed in free CST between the amino acid residues 175 to 190, which represents the loop region in the substrate-binding site, while in the CST–GC complex, residual fluctuation was minimized significantly, as interaction with the ligand may provide less room for fluctuation (Singh and Singh, 2024a). Among the four test complexes, CST–18alpha-glycyrrhetinic acid and CST–β-carotene showed relatively better stability between amino acid residues 180 and 190, while in the CST–lupeol complex, the fluctuation in this region was close to that of the free CST. Overall, the CST–18alpha-glycyrrhetinic acid and CST–β-carotene complexes showed residual fluctuation close to that of the CST–GC complex, which is again a good indication of the competitive inhibition of CST. The CST–lupeol complex was found to be least stable at the residual level.

#### 3.4.2 Structural compactness with Rg, SASA, and hydrogen bonding analysis

**FIGURE 4:**
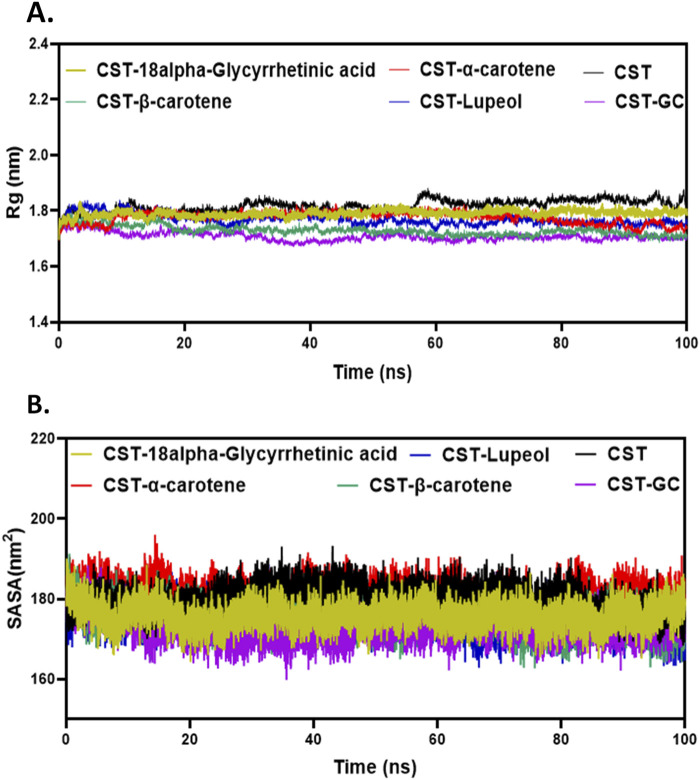
Structural compactness analysis of CST in the CST–18alpha-glycyrrhetinic acid, CST–α-carotene, CST–β-carotene, and CST–lupeol complexes. Time evolution of **(A)** Rg values and **(B)** SASA values during the simulation.

The next level of screening was to evaluate changes in the size or structural compactness of the protein in the presence of ligands during the simulation by measuring Rg of the protein–ligand complex (Lobanov et al., 2008; Rampogu et al., 2022; Funari et al., 2022). Rg also estimates the flexibility of the protein under complex formation by comparing it with Rg of either the free protein or the substrate-bound protein. The larger Rg in the free CST indicated lesser compactness and greater flexibility, whereas the smaller Rg in the CST–GC complex indicated greater compactness and rigidity in the protein structure in the presence of the substrate. As depicted in Figure 4A, a similar trend was visible with inhibitor binding. Of the four, the Rg of the CST–β-carotene complex was most closely related to the Rg of the protein–substrate complex. In the CST–18alpha-glycyrrhetinic acid complex, structural compactness of the protein was between that of free CST and the CST-GC complex, suggesting that the presence of the ligand slightly compressed the protein structure. The Rg values of the CST–α-carotene and CST–lupeol complexes were found to be between the Rg of the free CST and the Rg of the CST-GC complex, also suggesting that the protein structure takes on stiffness and compactness in the presence of these ligands (Figure 4A).

The SASA measures the solvent-accessible surface area of the free protein or protein in the protein–ligand complex. It basically calculates the surface area of molecules that is exposed to solvent molecules (Alrouji et al., 2023; Durham et al., 2009; Ali et al., 2014; Savojardo et al., 2021). The SASA analyzes how parts of the protein come into contact with the solvent over the simulation time. The average SASA range for the selected compounds was between 170 and 180 nm^2^, which falls between the range of the SASA of free CST (180.1 nm^2^) and that of CST complexed with the substrate (173.07 nm^2^) (Singh and Singh, 2024a). The average SASA of the CST–18alpha-glycyrrhetinic acid, CST–α-carotene, CST–β-carotene, and CST–lupeol complex was 175.83 nm^2^, 180.89 nm^2^, 175.38 nm^2^, and 175.30 nm^2^, respectively (Figure 4B). Of the four hits, the SASA of CST–18alpha-glycyrrhetinic acid, CST–β-carotene, and CST–lupeol falls within the expected range of the free CST and CST–substrate complex, suggesting better compactness of the protein in the presence of these ligands with relatively lesser unwanted solvent accessibility. The SASA of the CST–α-carotene complex was close to the SASA of free CST, suggesting that ligand binding has a negligible impact on the solvent accessibility of the protein structure. Overall, the SASA of the protein–ligand complex of the selected compounds showed no major changes in the exposed protein structure after protein–ligand binding and maintained the natural structural integrity of the protein.

**FIGURE 5:**
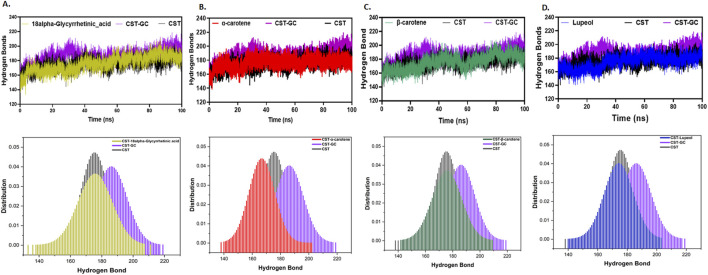
Time evolution of intramolecular hydrogen bond analysis in CST in **(A)** CST–18alpha-glycyrrhetinic acid, **(B)** CST–α-carotene, **(C)** CST–β-carotene, and **(D)** CST–lupeol complexes. The lower panel represents the probability distribution function (PDF) plot of each complex.

Furthermore, intramolecular hydrogen bonding analysis was vital for understanding the stability of the protein–ligand complex as ligand binding impacts the overall protein intramolecular dynamics (Pace et al., 2014; Hubbard and Haider, 2010). Compared to the free CST protein, substrate binding to the substrate binding site in CST slightly increased the intramolecular hydrogen bonding, suggesting that when binding to the active site, the substrate pushed the protein structure inward, facilitating the proximity of atoms and, thus, facilitated additional contacts at the intramolecular level. Among the protein–ligand complexes of the selected compounds, the CST–18alpha-glycyrrhetinic acid, CST–β-carotene, and CST–lupeol complexes showed a negligible impact on the intramolecular hydrogen bond dynamics of the CST protein, suggesting the stability of protein intramolecular dynamics with ligand binding (Figures 5A, C, D). Probability distribution function (PDF) plots show good consistency of these three complexes. The CST–α-carotene complex was found to be the least stable and most deviated complex, and the protein structure in this complex even showed negatively more flexibility than that of the free CST (Figure 5B).

Thus, at the structural compactness level, the CST–18alpha-glycyrrhetinic acid, CST–β-carotene, and CST–lupeol complexes were found to be relatively more stable than the CST–α-carotene complex. In the presence of these three compounds, the CST protein underwent the expected natural stiffness and compactness phenomenon, as observed in the case of protein–substrate binding.

#### 3.4.3 Principal component analysis

**FIGURE 6:**
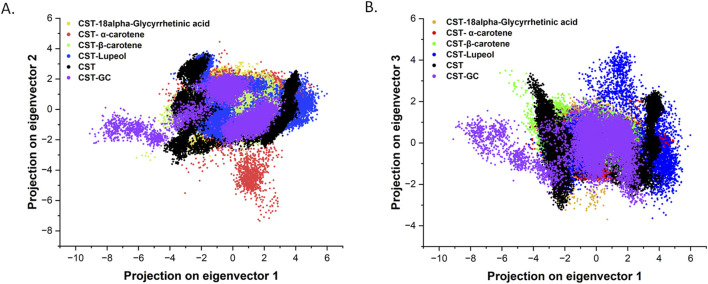
Principal component analysis of conformational projections of free CST and its complexes substrate (violet), 18alpha-Glycyrrhetinic acid (dark yellow), α-carotene (red), β-carotene (light green), and Lupeol (blue). **(A)** Projection of the first two eigenvectors 1 and 2 **(B)** projections of first three eigenvectors and plot between PC1 and PC3.

The specific catalytic action of each protein is executed through coordinated and collective atomic motion, which determines the stability of the protein (Zare et al., 2024; Yuan et al., 2024). PCA is widely used to analyze the atomic-level conformational changes through ligand binding. Basically, the principal components are the dominant mode of motion of the system that determine the structural and dynamic properties of the protein–ligand complex (Zare et al., 2024; Alrouji et al., 2023). In this study, principal components for the four selected CST–ligand complexes were largely determined by the first two (PC1 and PC2) and the first three eigenvectors (PC1, PC2, and PC3), which reflected the overall dynamics of the molecular subspace of the CST protein bound with the selected ligands (Figures 6A, B). With eigenvector 1 spanning between −3.3 and 4.5, eigenvector 2 between −3.07 and 3.09, and eigenvector 3 between −3.23 and 4.26, the CST–18alpha-glycyrrhetinic acid complex showed better compactness among the four. In the CST–β-carotene complex, eigenvector 1 covers −6.18 to 3.87, eigenvector 2 was between −3.3 and 2.7, and eigenvector 3 was between −1.7 and 3.4. With eigenvector 1 valued between −4.6 and 5.1 nm, eigenvector 2 from −7.3 to 4.1, and eigenvector 3 from −2.0 to 2.7 nm, the CST–α-carotene complex was found to be the most dispersed among the four, followed by the CST–lupeol complex, of which eigenvector 1 ranged between −4.3 and 5.9, eigenvector 2 from −2.6 to 3.8 nm, and eigenvector 3 from −3.6 to 4.6. In the first two eigenvectors, CST–18alpha-glycyrrhetinic acid, CST–β-carotene, and CST–lupeol complexes fell within the expected range of free CST and CST complexed with its substrate (GC) and showed relatively less motion and higher stability, while the CST–α-carotene complex was found to be the most diverted and less stable complex (Figure 6A). Subsequently, comparing the results of the first three principal components, we found that all four complexes primarily fall within the expected range of the free CST and CST–substrate complex, with slight diversions observed in the CST–lupeol complex (Figure 6B). Thus, in both sets of eigenvectors, CST–18alpha-glycyrrhetinic acid and CST–β-carotene demonstrated relatively better stability because their principal components occupied a smaller subspace. Thus, these findings further strengthen the candidature of 18alpha-glycyrrhetinic acid and β-carotene as inhibitors for CST.

#### 3.4.4 Free energy landscape analysis

**FIGURE 7:**
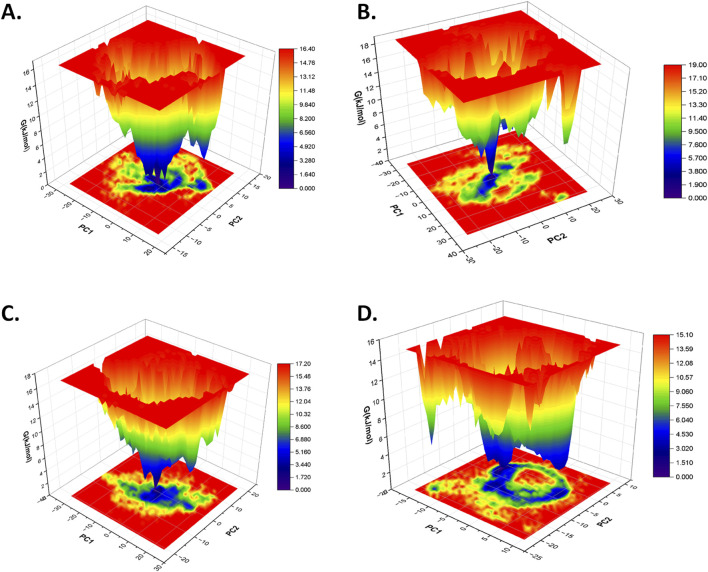
Free energy landscape of **(A)** CST–18alpha-glycyrrhetinic acid, **(B)** CST–α-carotene, **(C)** CST–β-carotene, and **(D)** CST–lupeol complexes.

The free energy landscape (FEL) analysis is primarily applied to understand the folding pattern of proteins and the impact of ligand binding on the structure of the protein (Plattner and Noé, 2015; Lau and Roux, 2007; Khan et al., 2019; Maisuradze et al., 2010). In this study, the first two eigenvectors were used from the principal component analysis to generate the FEL plot of each protein–ligand complex based on the dominant and stable conformation during a simulation of 100 ns. In contrast to the free CST, which showed relatively increased conformational space with global minima of 16.60 kJ/mol for attaining a stable structure, the conformational space of CST-GC complex slightly shifted with global minima of 17.10 KJ/mol (Singh and Singh, 2024a). In this study, in the spectrum of the free energy landscape, dark blue signified the most energetically favored region, and green depicted the moderately favored region, where the conformation of the protein could maintain its stability, while yellow represented the relatively unfavorable region and high energy state. According to the FEL plot, CST–18alpha-glycyrrhetinic acid, CST–β-carotene, and CST–lupeol complexes showed a wider blue zone with a global minima of 16.40 kJ/mol, 17.20 kJ/mol, and 15.10 kJ/mol, respectively (Figures 7A, C, D). The global minima of CST–18alpha-glycyrrhetinic acid and CST–β-carotene complexes were within the range of the global minima of the free CST (16.60 kJ/mol) and CST-GC complex (17.10 kJ/mol) (Singh and Singh, 2024a), thus showing potential for competitive inhibition in terms of energy requirement for binding. Among the four hits, the FEL of the CST–α-carotene complex with a global minima of 19.00 kJ/mol was found to have the least stable protein–ligand interaction (Figure 7B). Overall, the free energy landscape of the protein–ligand complexes provided a valuable insight into the interaction potential of the ligand and the stability of the protein in its bound form.

#### 3.4.5 Dynamic cross-correlation matrix analysis

**FIGURE 8:**
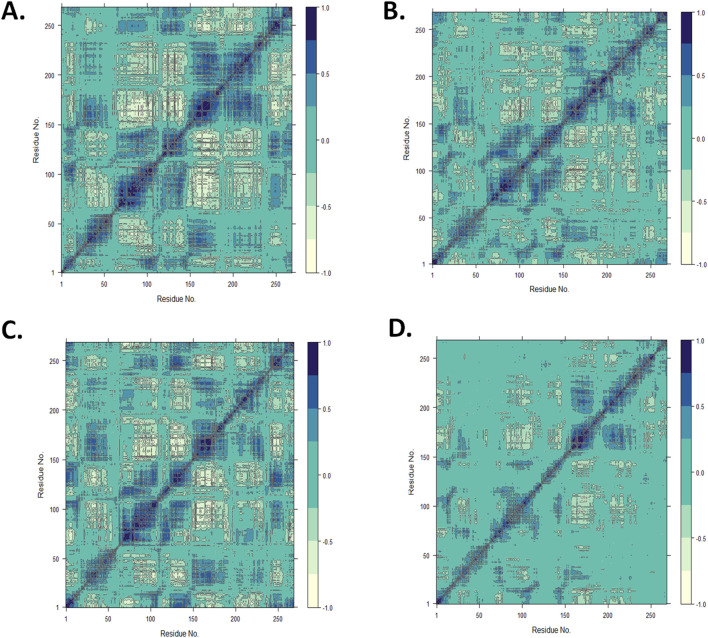
Dynamic cross-correlation map for the protein complexed with **(A)** 18alpha-glycyrrhetinic acid, **(B)** α-carotene, **(C)** β-carotene, and **(D)** lupeol, using the Bio3D package in RStudio.

Using the MD trajectory data, a DCCM analysis was performed to understand the dominant correlation network of amino acid residues in the CST protein in the presence of ligands (Du et al., 2010; Avti et al., 2022). The dynamic correlation mapping of protein residues in the presence of different ligands indicated the impact of the ligand on the overall protein structure conformational stability. For the DCCM study of the four complexes, the MD simulation structure in the last 10 ns was considered to obtain an insight into the protein–ligand complex. In the 2D matrix of DCCM analysis, the dark blue region represents the highly correlated motion of residues in the positive direction, the white region represents the highly anti-correlated motion of residues, and the no-color region indicates no correlation in the motion of the residue. Protein residues in the CST–18alpha-glycyrrhetinic acid complex were considered highly correlated because they occupied a wider area of dark blue (Figure 8A). The protein residues in the CST–β-carotene complex showed a relatively better residue correlation than that in the CST–α-carotene complex (Figures 8B, C). The residues in the CST–lupeol complex were found to have the least correlated residues as the dark blue region is relatively sparse among the four protein–ligand complexes, and the large area in this complex is uncolored, indicating no significant correlation and reduced relation with the target protein (Figure 8D). Overall, the correlated motion of the residue analyzed in this study showed the quality of the protein–ligand complex. The DCCM analysis of residues bound to 18alpha-glycyrrhetinic acid revealed the strongest correlation with the protein among all four complexes, thus indicating a strong protein–ligand interaction. In line with other MD trajectory analysis results, β-carotene also showed positive results in the DCCM analysis, showing a strong correlation with protein residues.

### 3.5 Post-MD protein–ligand interaction pose analysis

**FIGURE 9:**
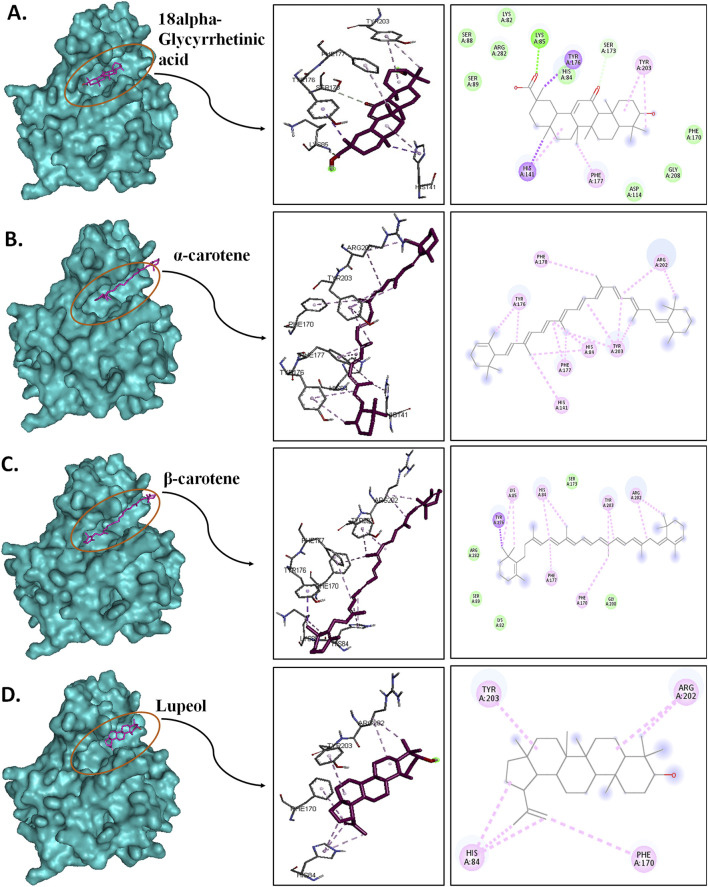
Post-MD interaction analysis of protein–ligand complexes with a surface view; 3D and 2D protein–ligand interaction of **(A)** CST–18alpha-glycyrrhetinic acid, **(B)** CST–α-carotene, **(C)** CST–β-carotene, and **(D)** CST–lupeol. Dark green, violet, light pink, and light green represent conventional hydrogen bonds, pi–sigma bonds, pi–alkyl bonds, and van der Waals interactions, respectively.

**FIGURE 10:**
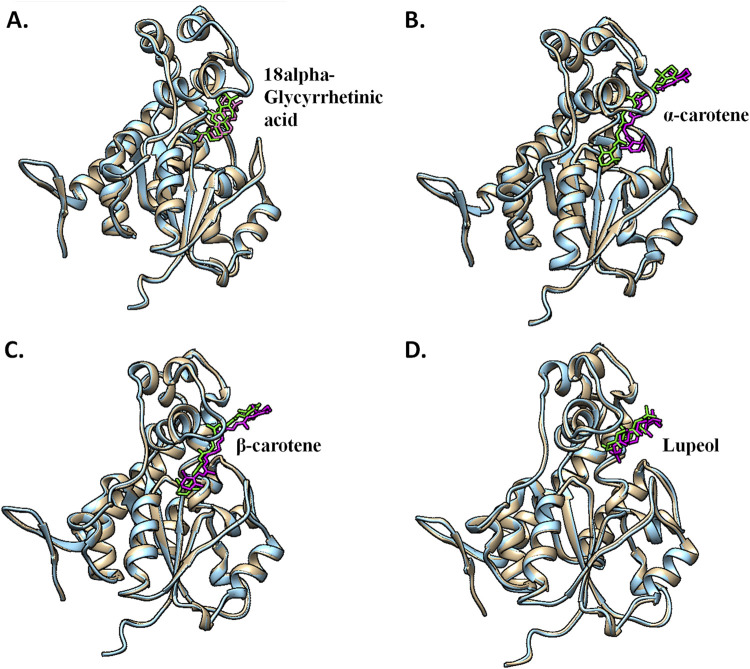
Pre- and post-MD structural alignment of **(A)** CST–18alpha-glycyrrhetinic acid, **(B)** CST–α-carotene, **(C)** CST–β-carotene, and **(D)** CST–lupeol complexes. Brown indicates the pre-MD CST complex with green ligands, and blue depicts the post-simulation protein complex with magenta ligands.

Post-simulation interaction analysis was an attempt to understand the binding pattern of the selected compounds under a dynamic aqueous environment, where the protein was free to take its most stable state. Of the four hits, 18alpha-glycyrrhetinic acid successfully retained its interaction with key residues, as well as its orientation and positioning in the binding pocket of the protein. In the CST–18alpha-glycyrrhetinic acid complex, as in the docked conformation, C=O of the carboxylic group formed a hydrogen bond with Lys85 in the polar site. In the middle of the binding pocket, His84 and His141 flank the compound on both sides, and hydrogen bond formation took place with His84, while Tyr203 at the aromatic side interacted with the compound through two pi–alkyl bonds (Figure 9A). Additionally, Ser173, as in the docked conformation, retained van der Waals interaction with the C=O group of the third aromatic ring of the compound. Thus, the CST–18alpha-glycyrrhetinic acid complex showed a strong protein–ligand interaction. During simulation, β-carotene also largely maintained a similar orientation in the binding pocket, with a slight change in the interaction pattern. Lys85 and Tyr176 in the left polar region, His84 and Phe177 in the middle, and Tyr203, Phe170, and Arg202 at the right end of the binding pocket determined the positioning of β-carotene in the binding pocket (Figure 9C). In contrast to the similarity in the docked conformation of CST–α-carotene and CST–β-carotene complexes, α-carotene was found to be the least stable in the binding pocket during the simulation. The isomeric aromatic ring of α-carotene shifted outward from the polar region away from Lys85, which was a key interacting residue in the docked conformation (Figure 9B). The simulation-led structural shift is shown in pre- and post-MD aligned complexes (Figure 10B). As in the docked conformation, the CST–lupeol complex maintained its position in the binding pocket, occupying mostly the right side of the binding pocket. Due to its relatively small size, lupeol left major room for ligand flexibility, which might not be suitable for competitive inhibition (Figure 9D). The distance of most of the pi-interactions was relatively short in both the CST–18alpha-glycyrrhetinic acid and CST–β-carotene-simulated complexes than their respective docked complexes, suggesting the strongest binding potential of 18alpha-glycyrrhetinic acid and the second strongest binding potential of β-carotene in the protein-binding pocket in the dynamic environment (Tables 3 and 4). Therefore, based on the interaction pattern analysis and alignment of pre- and post-MD complexes, 18alpha-glycyrrhetinic acid and β-carotene were found to be suitable candidates for further reverse pharmacophore analysis.

**TABLE 4:** Non-bonded interaction in the simulated protein–ligand complex.

Sl. no.	Compound	Residue in contact	Bond type	Distance (Å)
1	18alpha-Glycyrrhetinic acid	Lys85	Conventional hydrogen bond	1.90
		His84	Conventional hydrogen bond	3.50
		Tyr176	Pi–sigma	5.03
		His141	Pi–sigma	4.23
		Ser173	Van der Waals	4.24
		Phe177	Pi–alkyl	5.86
		Phe177	Pi–alkyl	5.30
		Tyr203	Pi–alkyl	5.81
		Tyr203	Pi–alkyl	7.31
2	α-Carotene	His84	Pi–alkyl	5.08
		His84	Pi–alkyl	6.02
		His141	Pi–alkyl	5.80
		Tyr176	Pi–alkyl	6.93
		Tyr176	Pi-Alkyl	5.93
		Phe170	Pi–alkyl	6.23
		Phe177	Pi–alkyl	4.98
		Phe177	Pi–alkyl	6.78
		Arg202	Pi–alkyl	4.01
		Tyr203	Pi–alkyl	4.80
		Tyr203	Pi–alkyl	5.19
		Tyr203	Pi–alkyl	5.38
3	β-Carotene	Lys85	Pi–alkyl	4.21
		Lys85	Pi–alkyl	4.79
		His84	Pi–alkyl	4.72
		His84	Pi–alkyl	4.83
		Phe170	Pi–alkyl	5.91
		Tyr176	Pi–sigma	4.84
		Phe177	Pi–alkyl	6.53
		Arg202	Pi–alkyl	3.84
		Arg202	Pi–alkyl	5.54
		Tyr203	Pi–alkyl	3.98
		Tyr203	Pi–alkyl	4.93
4	Lupeol	His84	Pi–alkyl	5.47
		His84	Pi–alkyl	5.68
		His84	Pi–alkyl	5.98
		Phe170	Pi–alkyl	5.67
		Tyr203	Pi–alkyl	6.29
		Arg202	Pi–alkyl	4.64
		Arg202	Pi–alkyl	7.69

### 3.6 Reverse pharmacophore mapping and cross-target identification

Reverse pharmacophore mapping for potential cross-target identification of the CST bioactive compounds (18alpha-glycyrrhetinic acid and β-carotene) was performed through PharmMapper, which compared the pharmacophore of the active compounds with the pharmacophore models of 300 proteins deposited in the database (Wang et al., 2017; Liu et al., 2010). Based on their fitness score cutoff ≥5.0, the key cross-targets were identified. Table 5 provides the details of the cross-target interactions of 18alpha-glycyrrhetinic acid and β-carotene. The two potential targets of 18alpha-glycyrrhetinic acid were corticosteroid 11-beta-dehydrogenase isozyme 1 and estradiol 17-beta-dehydrogenase 1, and positively, none of these are associated with the brain, while in the case of β-carotene, potential cross-targets are transthyretin, cellular retinoic acid-binding protein 2, and retinol-binding protein 4 (RBP4), among which transthyretin and RBP4 are associated with the brain.

**TABLE 5:** Reverse pharmacophore mapping for identifying potential targets for the best-performing compounds.

Compounds	Proteins identified in PharmMapper	PDB ID	Disease	No. of pharmacophore features	Fitness score
18alpha-Glycyrrhetinic acid	Corticosteroid 11-beta-dehydrogenase isozyme 1	2BEL	Polycystic ovary syndrome (PCOS)	10	5.383
	Estradiol 17-beta-dehydrogenase 1	1JTV	None	8	5.142
β-Carotene	Transthyretin	1RLB	Amyloidosis type 1 (AMYL1)	10	5.937
	Cellular retinoic acid-binding protein 2	1CBS	None	10	5.517
	Retinol-binding protein 4	1RBP	Night vision problems	8	5.081

## 4 Discussion

Metachromatic leukodystrophy is one of the critical neuropathological conditions that arise due to the accumulation of sulfatides over neurons, which leads to the development of white matter abnormalities and, thus, interrupt the proper processing of communication signals throughout the central and peripheral nervous systems. In the era of bioinformatics advancement, the option of developing an oral drug as a part of substrate reduction therapy is emerging as a new strategy in single-gene disorders including MLD. To counter the major existing challenge in SRT, i.e., the lack of the availability of the three-dimensional structure of the target protein, *cerebroside sulfotransferase*, our group successfully developed a 3D homology model of the protein, which was used as a receptor for screening of compounds in this study. In the present study, the substrate-binding site of the protein was used for grid generation and protein–ligand interaction study. For this study, four potential herbs of Medhya Rasayana were considered for screening their phytoconstituents as these herbs are well-regarded as memory enhancers. Initially, based on a binding score cutoff of ≤−7.5 kcal/mol, the top 15 compounds were selected for performing pharmacokinetics studies to eliminate false positives. Along with showing no significant toxicity under ADMET analysis, the four potential compounds—18alpha-glycyrrhetinic acid, α-carotene, β-carotene, and lupeol—showed high blood–brain barrier permeability, which was considered a critical parameter for the selection of inhibitors as the target’s physical location is the brain, and it was important to select those compounds that can cross the blood–brain barrier smoothly.

The interaction analysis of these four compounds in pre- and post-MD showed interesting outcomes, which became a basis for the identification of the most potent compounds for CST among the four compounds. In the pre-MD protein–ligand complex, the protein was rigid, and flexible ligands attempted to adjust in the active site. However, during MD, the protein was subjected to an aqueous environment, which provided enough flexibility to the protein to adjust itself in the best possible way to provide maximum stability to its complex formation with the ligand. Among the four complexes, 18alpha-glycyrrhetinic acid showed a better interaction with the CST active site while maintaining its position throughout the simulation (Figure 10A). The carboxylic acid end of the compound facilitated the orientation of compounds toward the polar region dominated by Lys85, Lys82, and Tyr176. The middle part of the compound was positioned exactly in the middle of the active site and flanked by His84 and His141 on both sides. Additionally, the oxygen atom at the middle of the compound facilitated its tight interaction in the active site. Phe170, Phe177, and Tyr203 were key residues in the aromatic site and interacted with the compound via a pi–alkyl interaction. In the pre-MD interaction study, α-carotene and β-carotene showed a similar interaction pattern, with minor differences at their isomeric ends. However, after MD, β-carotene maintained its position in the active site (Figure 10C), while α-carotene deviated from its initial position toward outside, as shown in Figure 10B. Based on active site occupancy criteria, lupeol, despite maintaining its position in the active site throughout the simulation, was regarded as less potent as it left enough space in the active site for other ligands to either cross-contaminate the active site or replace it if the other ligand is available in sufficient concentration (Figure 10D). Therefore, lupeol was considered less suitable to satisfy the competitiveness criteria.

The in-depth trajectory analysis of the selected compounds provided sufficient information for understanding the above structural changes. The RMSD of four ligands showed stability in terms of overall structural deviation within the stipulated simulation time. The residual-level fluctuation was sharpened in the RMSF study, where the fluctuation was more pronounced in lupeol, followed by α-carotene. The CST–18alpha-glycyrrhetinic acid and CST–β-carotene complexes showed better residual stability. Regarding structural compactness parameters including Rg and SASA, the protein in all four complexes largely maintained its structural compactness within the range of the free CST and CST–substrate complex, while at intramolecular hydrogen-bond formation-based structural compactness criteria, the protein in the CST–α-carotene complex showed least compactness among the four complexes. Furthermore, in PCA, the deviation of α-carotene and lupeol was more pronounced, while CST–18alpha-glycyrrhetinic acid and CST–β-carotene were relatively better confined with occupying minimal subspace. A similar trend was also observed in the free energy landscape analysis; among the four, only 18alpha-glycyrrhetinic acid and β-carotene showed global minima between the range of the free CST and CST–substrate complex, while α-carotene was far above the range, and lupeol was found far below the range. In the dynamic cross-correlation map, the CST–lupeol complex was found to be least correlated. In the correlation matrix, the CST–18alpha-glycyrrhetinic complex was found to be the best correlated complex among the four, followed by the CST–β-carotene complex. Thus, the overall trajectory analysis represented the CST–α-carotene and CST–lupeol complexes as lesser stable complexes, hence excluded from further studies.

Reverse pharmacophore mapping was carried out to rule out the possibilities of cross-contamination with other nervous system proteins. With no potential neurodegenerative disease link target, 18alpha-glycyrrhetinic acid can be considered the most potent and specific drug candidate against CST, while in the case of β-carotene, transthyretin and RBP4 may act as cross-targets in the brain (Pinheiro et al., 2022; Ishii et al., 2019). Cross-binding of β-carotene to transthyretin may cause β-amyloid deposition, leading to neuropathological conditions. In the central nervous system, RBP4 binds and inhibits transthyretin and, thus, regulates the breakdown of amyloid-β, whereas the cross-interaction of β-carotene with RBP4 may reduce the β-amyloid deposition by restricting the interaction of RBP4 with transthyretin (Pinheiro et al., 2022). To understand the merit of the existing discrepancies with the cross-target interactions of β-carotene with other proteins, a strong recommendation is made to further validate the result through experimental data.

Thus, the combinatorial high-throughput *in silico* screening procedures conducted in this study suggested the overall stability of 18alpha-glycyrrhetinic acid and β-carotene inside the active site pocket of the CST protein. Although 18alpha-glycyrrhetinic acid was found to be the best performer throughout the screening while maintaining the overall integrity of the protein conformation during complex formation in a dynamic simulation environment, β-carotene can also be considered for further studies as the second-best drug candidate as it also showed strong binding affinity in the binding site pocket of CST. Thus, the application of molecular docking, ADMET analysis, and simulation-led trajectory analysis provided a thorough understanding of the overall stability of the protein–ligand interaction. However, despite the multiple levels of stringent screening done in this study, there is no ruling out the possibility of failure of the proposed drug candidates at the experimental level. Therefore, the potency and specificity determined in this study need further validation through *in vitro* enzymatic, cell line studies and subsequent *in vivo* validation using animal models to complete the preclinical drug discovery validation process to obtain a potent drug candidate for the clinical trial of future marketed drugs. Additionally, since these drug candidates are originated from medicinal plants, they can also open the door for combinatorial substrate reduction therapy where potent drugs can combine with the hydroalcoholic extract of the whole plant to minimize any form of side effects of potent compounds while maintaining the potency via optimizing the dosage combination. Thus, the idea is to develop the safest possible oral medication for metachromatic leukodystrophy, which is one of the most fatal genetic diseases in children.

## 5 Conclusion

*Cerebroside sulfotransferase* is identified as an attractive target protein to develop substrate reduction therapy as a new therapeutic intervention in the field of metachromatic leukodystrophy. This study aimed to identify a potent and selective inhibitor for CST inhibition using a multipronged virtual screening approach using a library of phytoconstituents of popular herbs of Medhya Rasayana. The screening of key neuroprotective herbs of Medhya Rasayana is an important strategy to identify potential neuroprotective inhibitors of CST. The phytoconstituents are natural compounds and are more adaptable to the natural system. Identifying potent drug candidates will further strengthen the potency of Ayurvedic herbs by applying the combinatorial strategy of treatment while minimizing any negative impact of the high concentration of drug candidates. In this study, 18alpha-glycyrrhetinic acid was identified as the most potent and specific inhibitor of CST, while β-carotene was found to be the second-most potent inhibitor of CST. Thus, the virtual screening approach applied in this study is a targeted approach that saved time and resource compared to the traditional approach of testing random chemicals through enzyme technology or applying a chemical synthesis approach for testing new drug entities as inhibitors. The MD simulation provided an insight into the binding pattern and orientation of compounds in the active site by providing knowledge about the most dominant mode of the motion responsible for determining the potency and selectivity of compounds. This information would be beneficial for designing new compounds based on the amino acid composition of the active site pocket. Thus, the findings of the present study narrow down the large pool of datasets and provide a direction for further *in vitro* and *in vivo* studies.

## Data Availability

All data generated or analyzed during this study can be found in this published article and its supplementary file, and are also available from the corresponding author upon reasonable request.
