# Supplementary material for: Exploration of phytoconstituents of Medhya Rasayana herbs to identify potential inhibitors for cerebroside sulfotransferase through high-throughput screening
Source: Front Mol Biosci. 2024 Oct 9;11:1476482. doi: 10.3389/fmolb.2024.1476482 (PMC11500077; doi:10.3389/fmolb.2024.1476482)
Supplement: Supplementary file 1 [file Table1.DOCX]

**Supplementary information**

**Exploration of phytoconstituents of Medhya Rasayana herbs to identify potential inhibitors for *Cerebroside sulfotransferase* through high-throughput screening.**

**Nivedita Singh^a^* and Anil Kumar Singh^a^**

^a^Department of Dravyaguna, Faculty of Ayurveda, Institute of Medical Sciences, Banaras Hindu University, Varanasi, Uttar Pradesh-221005, India

***Corresponding Author**

Dr. Nivedita Singh, Department of Dravyaguna, Faculty of Ayurveda, Institute of Medical Sciences, Banaras Hindu University, Varanasi, Uttar Pradesh-221005, India-221005, Email: niv234@gmail.com, s.nivedita@bhu.ac.in

**Table S1:** **81 phytoconstituents of *Centella asiatica***

| **Sl. No.** | **IMMPAT ID** | **Phytochemical Name** | **No. of conformation** | **Lowest free energy of binding in largest cluster** | **Number of conformations in largest cluster** |
| --- | --- | --- | --- | --- | --- |
|  | IMPHY000399 | beta-Bisabolene | 4 | -5.60 | 63 |
|  | IMPHY000583 | Asiaticoside | 74 | -4.56 | 6 |
|  | IMPHY000921 | alpha-Chamigrene | 1 | -5.80 | 100 |
|  | IMPHY001223 | 2,3-Dihydrobenzofuran | 6 | -5.20 | 57 |
|  | IMPHY001248 | quadranoside IV | 32 | -7.46 | 19 |
|  | IMPHY001658 | Thymol methyl ether | 3 | -4.87 | 83 |
|  | IMPHY002100 | Isothankunic acid | 8 | -8.33 | 51 |
|  | IMPHY003276 | hydrocotylin | 12 | -5.48 | 28 |
|  | IMPHY003482 | 4-Methoxybenzaldehyde | 5 | -4.83 | 63 |
|  | IMPHY003485 | Myrcene | 4 | -4.48 | 44 |
|  | IMPHY003495 | p-vinylguaicol | 9 | -4.52 | 48 |
|  | IMPHY003649 | 2-Heptenal | 7 | -4.43 | 39 |
|  | IMPHY003657 | asiatic acid | 12 | -8.23 | 39 |
|  | IMPHY003982 | gamma-Terpinene | 4 | -4.19 | 66 |
|  | IMPHY004187 | L-(+)-Arabinose | 16 | -3.79 | 26 |
|  | IMPHY004248 | Asiaticoside A | 77 | -4.40 | 4 |
|  | IMPHY004249 | asiaticoside a | 85 | -4.10 | 5 |
|  | IMPHY004388 | Kaempferol | 17 | -5.28 | 32 |
|  | IMPHY004597 | Rosmarinic acid | 47 | -7.60 | 8 |
|  | IMPHY004619 | Quercetin | 18 | -5.16 | 20 |
|  | IMPHY004660 | Luteolin | 14 | -6.14 | 32 |
|  | IMPHY004661 | Apigenin | 11 | -5.94 | 26 |
|  | IMPHY005115 | Ursolic acid lactone | 1 | -8.54 | 100 |
|  | IMPHY005471 | Myricetin | 19 | -4.85 | 17 |
|  | IMPHY005549 | 2-Octenal | 10 | -4.40 | 27 |
|  | IMPHY006347 | Hexanal | 4 | -4.15 | 62 |
|  | IMPHY006362 | Ascorbic acid | 25 | -3.87 | 25 |
|  | IMPHY006469 | 2-Methyl-2-butanol | 5 | -3.75 | 57 |
|  | IMPHY006612 | Corosolic acid | 12 | -7.50 | 31 |
|  | IMPHY006717 | Anthrone | 2 | -5.63 | 89 |
|  | IMPHY006802 | madasiatic acid | 11 | -7.98 | 32 |
|  | IMPHY006932 | Cuparene | 2 | -5.40 | 89 |
|  | IMPHY006952 | m-Xylene | 3 | -4.80 | 74 |
|  | IMPHY007041 | Furfural | 3 | -4.20 | 96 |
|  | IMPHY007186 | Heptanal | 7 | -4.42 | 48 |
|  | IMPHY007222 | Pentanal | 3 | -3.71 | 69 |
|  | IMPHY007228 | Terminoloside | 83 | -3.93 | 6 |
|  | IMPHY010179 | (-)-beta-Chamigrene | 1 | -5.86 | 100 |
|  | IMPHY010286 | Gulonic acid | 10 | -4.68 | 45 |
|  | IMPHY010964 | Dammarane | 4 | -8.90 | 63 |
|  | IMPHY010995 | Toluene | 2 | -4.42 | 81 |
|  | IMPHY011586 | germacrane d | 1 | -6.25 | 100 |
|  | IMPHY011609 | alpha-Carotene | 3 | -7.90 | 96 |
|  | IMPHY011626 | Bayogenin | 8 | -8.63 | 62 |
|  | IMPHY011632 | Farnesol | 24 | -5.08 | 13 |
|  | IMPHY011643 | alpha-Terpinene | 4 | -4.66 | 58 |
|  | IMPHY011658 | beta-Farnesene | 6 | -5.12 | 65 |
|  | IMPHY011761 | Humulene | 2 | -5.63 | 95 |
|  | IMPHY011797 | Oleic acid | 17 | -5.93 | 27 |
|  | IMPHY011844 | Chlorogenic acid | 32 | -7.19 | 16 |
|  | IMPHY011880 | Ursolic acid | 2 | -9.28 | 99 |
|  | IMPHY011957 | (+)-delta-Cadinene | 1 | -6.26 | 100 |
|  | IMPHY011961 | iso-chlorogenic acid | 33 | -7.50 | 23 |
|  | IMPHY012003 | Betulinic acid | 5 | -9.23 | 63 |
|  | IMPHY012010 | Ginsenosides | 6 | -8.44 | 50 |
|  | IMPHY012058 | Linalool | 10 | -4.37 | 30 |
|  | IMPHY012061 | alpha-Pinene | 2 | -5.81 | 51 |
|  | IMPHY012147 | beta-Pinene | 2 | -6.13 | 61 |
|  | IMPHY012160 | alpha-Terpineol | 12 | -4.37 | 36 |
|  | IMPHY012227 | Madecassic acid | 20 | -8.01 | 30 |
|  | IMPHY012289 | epi-Globulol | 2 | -6.30 | 96 |
|  | IMPHY012402 | Campesterol | 9 | -8.57 | 32 |
|  | IMPHY012589 | β-sesquiphellandrene | 4 | -5.91 | 73 |
|  | IMPHY012654 | Nerol | 11 | -4.58 | 38 |
|  | IMPHY012667 | Caryophyllene oxide | 2 | -6.11 | 99 |
|  | IMPHY012721 | Isoquercitrin | 61 | -4.89 | 9 |
|  | IMPHY014396 | Quercetin-3-glucoside | 58 | -4.90 | 10 |
|  | IMPHY014824 | Astragalin | 50 | -4.87 | 11 |
|  | IMPHY014831 | beta-Caryophyllene | 3 | -5.75 | 97 |
|  | IMPHY014836 | beta-Sitosterol | 7 | -8.67 | 45 |
|  | IMPHY014842 | Stigmasterol | 11 | -8.47 | 28 |
|  | IMPHY014852 | Camphene | 2 | -4.67 | 56 |
|  | IMPHY014893 | D-Glucose | 13 | -4.43 | 69 |
|  | IMPHY014923 | Geraniol | 14 | -4.58 | 50 |
|  | IMPHY014988 | Limonene | 3 | -4.52 | 59 |
|  | IMPHY015022 | Nerolidol | 17 | -5.00 | 27 |
|  | IMPHY015056 | L-Rhamnose | 5 | -4.24 | 89 |
|  | IMPHY015123 | alpha-Copaene | 1 | -6.78 | 100 |
|  | IMPHY015637 | 3-Nonen-2-one | 12 | -4.71 | 28 |
|  | IMPHY016366 | 3-Hexen-2-one | 3 | -4.62 | 67 |
|  | IMPHY016460 | 5-nonen-2-one | 14 | -4.30 | 22 |

**Table S2:** **310 phytoconstituents of *Glycirrhiza glabra***

| **Sl. No.** | **IMMPAT ID** | **Phytochemical Name** | **No. of conformation** | **Lowest free energy of binding (kcal/mol) in the largest cluster** | **Number of conformations in largest cluster** |
| --- | --- | --- | --- | --- | --- |
|  | IMPHY000013 | Licoflavone B | 16 | -7.88 | 36 |
|  | IMPHY000053 | alphaalpha-dihydro-354-trihydroxy-5-isopentenylstilbene | 33 | -6.46 | 10 |
|  | IMPHY000060 | Myristic acid | 12 | -5.30 | 46 |
|  | IMPHY000151 | Glabroisoflavanone A | 4 | -7.28 | 82 |
|  | IMPHY000295 | Paeonol | 6 | -4.97 | 92 |
|  | IMPHY000308 | Hexadecane | 11 | -3.78 | 29 |
|  | IMPHY000318 | 3,3',5'-Trihydroxy-4-methoxy-5-prenylbibenzyl | 49 | -5.50 | 10 |
|  | IMPHY000375 | 3-Hydroxyglabrol | 42 | -6.89 | 15 |
|  | IMPHY000603 | Kanzonol Y | 61 | -5.17 | 7 |
|  | IMPHY000688 | 7-Methoxycoumarin | 8 | -5.40 | 31 |
|  | IMPHY000742 | Glyasperin D | 19 | -6.70 | 27 |
|  | IMPHY000754 | 6,8-diprenylorobol | 29 | -6.46 | 19 |
|  | IMPHY000765 | 1-Methoxyphaseollidin | 13 | -7.00 | 31 |
|  | IMPHY000776 | Licoricidin | 27 | -6.93 | 13 |
|  | IMPHY000791 | Gancaonin R | 56 | -5.68 | 7 |
|  | IMPHY000819 | 8-Prenylnaringenin | 18 | -6.21 | 38 |
|  | IMPHY000852 | Glabrene | 9 | -6.97 | 55 |
|  | IMPHY000862 | Isoflavan | 2 | -6.06 | 95 |
|  | IMPHY000863 | Glyinflanin B | 18 | -6.51 | 45 |
|  | IMPHY000864 | Glycyrin | 21 | -6.77 | 28 |
|  | IMPHY000871 | Gancaonin I | 15 | -6.57 | 19 |
|  | IMPHY001010 | Liquiritin apioside | 59 | -5.89 | 7 |
|  | IMPHY001239 | Shinpterocarpin | 2 | -7.05 | 96 |
|  | IMPHY001244 | Licoagrodione | 45 | -6.03 | 12 |
|  | IMPHY001245 | alphaalpha-dihydro-3534-tetrahydroxy-45-diisopentenylstilbene | 56 | -5.72 | 9 |
|  | IMPHY001246 | Carvacrol | 11 | -4.69 | 56 |
|  | IMPHY001262 | soyasaponin ii | 44 | -6.42 | 18 |
|  | IMPHY001308 | Retinol | 3 | -7.20 | 98 |
|  | IMPHY001315 | Flavanone | 4 | -6.09 | 76 |
|  | IMPHY001500 | Hispaglabridin B | 1 | -7.57 | 100 |
|  | IMPHY001586 | Isoglabrolide | 2 | -8.44 | 82 |
|  | IMPHY001688 | 2-Acetylpyrrole | 7 | -4.76 | 51 |
|  | IMPHY001793 | Glabroisoflavanone B | 6 | -6.34 | 88 |
|  | IMPHY001798 | Glabrocoumarin | 11 | -6.51 | 35 |
|  | IMPHY001806 | Liqcoumarin | 8 | -4.89 | 35 |
|  | IMPHY001806 | Liqcoumarin | 8 | -4.89 | 35 |
|  | IMPHY001866 | Glabrol | 28 | -7.46 | 22 |
|  | IMPHY001869 | Liquiritigenin | 12 | -6.11 | 25 |
|  | IMPHY001915 | Octadecane | 11 | -3.54 | 21 |
|  | IMPHY001923 | 4-Methylcoumarin | 3 | -5.35 | 91 |
|  | IMPHY001944 | Glychionide A | 32 | -8.41 | 25 |
|  | IMPHY002003 | 5-Methylfurfural | 3 | -4.72 | 66 |
|  | IMPHY002304 | liquoric acid | 2 | -10.22 | 95 |
|  | IMPHY002305 | 21alpha-Hydroxyisoglabrolide | 3 | -7.86 | 46 |
|  | IMPHY002667 | Pentadecanoic acid | 10 | -5.63 | 49 |
|  | IMPHY002720 | 2-Acetyl-5-methylfuran | 5 | -4.99 | 49 |
|  | IMPHY002750 | 2-Acetylfuran | 6 | -4.64 | 47 |
|  | IMPHY002787 | 1-Methylpyrrole-2-carboxaldehyde | 10 | -3.91 | 44 |
|  | IMPHY002803 | tetramethylpyrazine | 4 | -4.15 | 40 |
|  | IMPHY002825 | 2-(4-Methylphenyl)propan-2-ol | 13 | -4.25 | 38 |
|  | IMPHY002875 | Undecane | 8 | -4.00 | 26 |
|  | IMPHY002915 | Benzyl Alcohol | 6 | -4.40 | 32 |
|  | IMPHY002926 | 2,3-Butanediol | 4 | -3.54 | 36 |
|  | IMPHY002949 | 1-Butanol | 8 | -3.05 | 36 |
|  | IMPHY002958 | 2,3,5-Trimethylpyrazine | 5 | -4.53 | 47 |
|  | IMPHY002962 | Benzoic acid | 2 | -6.49 | 97 |
|  | IMPHY002982 | 7-Acetoxy-2-methylisoflavone | 8 | -6.84 | 30 |
|  | IMPHY003016 | Lauric acid | 9 | -5.29 | 27 |
|  | IMPHY003035 | Paratocarpin B | 30 | -7.64 | 19 |
|  | IMPHY003037 | Methoxsalen | 2 | -5.67 | 95 |
|  | IMPHY003104 | Decanoic acid | 8 | -5.43 | 24 |
|  | IMPHY003113 | p-Cresol | 5 | -4.58 | 55 |
|  | IMPHY003256 | 7-methoxy-2-methylisoflavone | 6 | -5.99 | 64 |
|  | IMPHY003283 | m-Cresol | 7 | -4.69 | 30 |
|  | IMPHY003296 | Piperitenone | 3 | -4.98 | 87 |
|  | IMPHY003301 | Octanoic acid | 4 | -5.97 | 69 |
|  | IMPHY003324 | (+)-Vestitol | 12 | -5.77 | 37 |
|  | IMPHY003340 | Acetoin | 9 | -4.15 | 49 |
|  | IMPHY003394 | onocerin | 8 | -9.50 | 67 |
|  | IMPHY003486 | Diacetone alcohol | 7 | -4.50 | 30 |
|  | IMPHY003490 | Coumarin | 6 | -6.08 | 46 |
|  | IMPHY003536 | Eugenol | 10 | -4.62 | 26 |
|  | IMPHY003537 | Tetradecanal | 20 | -3.74 | 22 |
|  | IMPHY003549 | Medicarpin | 6 | -5.62 | 31 |
|  | IMPHY003555 | o-Cresol | 9 | -4.82 | 42 |
|  | IMPHY003809 | echinatine | 23 | -3.86 | 27 |
|  | IMPHY004109 | Soyasaponin I | 50 | -5.96 | 10 |
|  | IMPHY004133 | 24 - hydroxy glycyrrhetic acid | 5 | -9.87 | 74 |
|  | IMPHY004235 | D-Glucuronic Acid | 4 | -5.79 | 78 |
|  | IMPHY004237 | Apiin | 74 | -6.06 | 6 |
|  | IMPHY004344 | Pratol | 8 | -6.01 | 42 |
|  | IMPHY004360 | Kumatakenin | 16 | -4.98 | 20 |
|  | IMPHY004362 | Licoagrone | 83 | -8.06 | 3 |
|  | IMPHY004365 | Licoricone | 25 | -5.73 | 16 |
|  | IMPHY004374 | Licochalcone a | 20 | -6.39 | 44 |
|  | IMPHY004388 | Kaempferol | 15 | -5.27 | 43 |
|  | IMPHY004395 | Licoflavone A | 12 | -6.75 | 32 |
|  | IMPHY004405 | Glycyrol | 13 | -6.30 | 26 |
|  | IMPHY004460 | soyasaponin | 57 | -4.89 | 10 |
|  | IMPHY004477 | 4'-O-Methylglabridin | 6 | -6.85 | 66 |
|  | IMPHY004522 | N-Acetylpyrrole | 5 | -4.43 | 64 |
|  | IMPHY004549 | Safrole | 10 | -4.92 | 53 |
|  | IMPHY004583 | Wighteone | 27 | -5.78 | 18 |
|  | IMPHY004586 | Texasin | 6 | -6.07 | 37 |
|  | IMPHY004595 | Licoisoflavone A | 27 | -6.47 | 25 |
|  | IMPHY004601 | Prunetin | 10 | -6.09 | 29 |
|  | IMPHY004619 | Quercetin | 18 | -5.06 | 15 |
|  | IMPHY004636 | Liquiritigenin 7-beta-D-glucopyranoside | 37 | -7.00 | 10 |
|  | IMPHY004643 | Genistein | 7 | -5.64 | 40 |
|  | IMPHY004661 | Apigenin | 13 | -5.94 | 25 |
|  | IMPHY004701 | Glyzarin | 9 | -6.03 | 74 |
|  | IMPHY004758 | Licocoumarone | 20 | -5.75 | 24 |
|  | IMPHY004759 | Liquiritin | 25 | -7.22 | 29 |
|  | IMPHY004868 | Hispaglabridin A | 10 | -7.00 | 51 |
|  | IMPHY004974 | Glabrone | 3 | -6.34 | 68 |
|  | IMPHY004981 | Docosyl caffeate | 69 | -3.42 | 4 |
|  | IMPHY004987 | Glycycoumarin | 24 | -5.96 | 29 |
|  | IMPHY004988 | Glycyrrhisoflavone | 22 | -6.46 | 22 |
|  | IMPHY005005 | Lupiwighteone | 21 | -6.03 | 31 |
|  | IMPHY005049 | Isolicoflavonol | 31 | -6.23 | 15 |
|  | IMPHY005051 | Isoliquiritin | 30 | -7.81 | 26 |
|  | IMPHY005055 | Isoononin | 23 | -6.74 | 23 |
|  | IMPHY005062 | Glyzaglabrin | 13 | -5.69 | 44 |
|  | IMPHY005381 | 1-methoxy-4-isopropyl-cyclohexane | 4 | -5.13 | 90 |
|  | IMPHY005401 | Uralsaponin B | 39 | -7.53 | 12 |
|  | IMPHY005428 | Bergapten | 3 | -5.29 | 68 |
|  | IMPHY005434 | Galangin | 14 | -5.02 | 33 |
|  | IMPHY005518 | 2-Acetyl-1-furfurylpyrrole | 4 | -5.42 | 59 |
|  | IMPHY005574 | 4-Hydroxychalcone | 10 | -5.68 | 25 |
|  | IMPHY005587 | Umbelliferone | 8 | -4.76 | 31 |
|  | IMPHY005638 | Licoagroside B | 58 | -5.89 | 10 |
|  | IMPHY005669 | Neoisoliquiritin | 38 | -6.23 | 26 |
|  | IMPHY005674 | 2-Furylacetone | 8 | -4.81 | 40 |
|  | IMPHY005730 | araboglycyrrhizin | 31 | -9.27 | 10 |
|  | IMPHY005731 | Apioglycyrrhizin | 24 | -7.61 | 18 |
|  | IMPHY005762 | Isoangustone A | 25 | -7.30 | 18 |
|  | IMPHY005811 | 2-Pentylfuran | 6 | -4.54 | 50 |
|  | IMPHY005863 | Glisoflavone | 22 | -6.71 | 47 |
|  | IMPHY005876 | Licoisoflavone B | 7 | -6.01 | 59 |
|  | IMPHY005893 | Licoflavonol | 16 | -5.96 | 49 |
|  | IMPHY005923 | 7-hydroxy-2-methylisoflavone | 5 | -6.07 | 86 |
|  | IMPHY005925 | Hydroxywighteone | 30 | -5.46 | 15 |
|  | IMPHY006083 | Glucoliquiritin apioside | 83 | -4.08 | 4 |
|  | IMPHY006145 | p-Cymene | 4 | -4.35 | 53 |
|  | IMPHY006193 | Glycerol | 13 | -3.32 | 69 |
|  | IMPHY006247 | Echinatin | 14 | -5.73 | 38 |
|  | IMPHY006347 | Hexanal | 4 | -4.19 | 64 |
|  | IMPHY006410 | Ethyl linolenate | 24 | -4.38 | 13 |
|  | IMPHY006428 | (R)-Lavandulol | 13 | -4.18 | 34 |
|  | IMPHY006486 | Squalene | 11 | -5.49 | 18 |
|  | IMPHY006489 | Isoliquiritigenin | 10 | -5.95 | 33 |
|  | IMPHY006550 | 2-isopropyl-5-methylphenol (Thymol) | 9 | -4.31 | 32 |
|  | IMPHY006552 | 2',4'-Dihydroxyacetophenone | 12 | -4.64 | 41 |
|  | IMPHY006595 | 1-(5-Methyl-2-furanyl)-1,2-propanedione | 8 | -4.73 | 36 |
|  | IMPHY006684 | Isoflavone | 3 | -5.90 | 71 |
|  | IMPHY006690 | Magnolol | 7 | -6.11 | 82 |
|  | IMPHY006696 | Methyleugenol | 4 | -4.67 | 73 |
|  | IMPHY006740 | Glabrolide | 4 | -8.23 | 86 |
|  | IMPHY006745 | Phaseollinisoflavan | 3 | -6.56 | 95 |
|  | IMPHY006776 | Licopyranocoumarin | 11 | -5.83 | 34 |
|  | IMPHY006894 | Ononin | 21 | -6.98 | 31 |
|  | IMPHY006944 | Estragole | 6 | -4.53 | 62 |
|  | IMPHY006951 | Eicosane | 12 | -4.84 | 23 |
|  | IMPHY006953 | 2,6-Dimethylpyrazine | 3 | -4.16 | 69 |
|  | IMPHY006967 | formetin | 11 | -5.83 | 31 |
|  | IMPHY006970 | Decanal | 17 | -3.50 | 27 |
|  | IMPHY006981 | Indole | 4 | -5.55 | 59 |
|  | IMPHY007006 | Furfuryl alcohol | 6 | -3.93 | 46 |
|  | IMPHY007041 | Furfural | 3 | -4.21 | 96 |
|  | IMPHY007058 | Maltol | 5 | -4.52 | 69 |
|  | IMPHY007068 | Undecanoic acid | 8 | -5.28 | 32 |
|  | IMPHY007076 | Undecanal | 20 | -3.63 | 20 |
|  | IMPHY007100 | 1-Dodecanol | 24 | -3.47 | 15 |
|  | IMPHY007169 | Pterocarpan | 2 | -5.55 | 88 |
|  | IMPHY007171 | 1-Hexanol | 11 | -3.94 | 30 |
|  | IMPHY007186 | Heptanal | 8 | -4.49 | 54 |
|  | IMPHY007195 | Heptanoic acid | 4 | -5.69 | 67 |
|  | IMPHY007196 | Pinocembrin | 11 | -5.54 | 28 |
|  | IMPHY007204 | Dodecanal | 18 | -3.88 | 17 |
|  | IMPHY007267 | beta-Terpinene | 3 | -4.63 | 57 |
|  | IMPHY007354 | Hexanoic acid | 2 | -5.62 | 53 |
|  | IMPHY007357 | Nicotinic acid | 4 | -6.14 | 69 |
|  | IMPHY007417 | Ethyl acetate | 4 | -3.50 | 89 |
|  | IMPHY007465 | Licochalcone C | 20 | -6.66 | 43 |
|  | IMPHY007499 | Licochalcone B | 17 | -6.04 | 21 |
|  | IMPHY007508 | Licoarylcoumarin | 17 | -5.62 | 29 |
|  | IMPHY007620 | 1-Octanol | 17 | -3.55 | 20 |
|  | IMPHY007720 | Methyl ethyl ketone | 4 | -3.41 | 77 |
|  | IMPHY007958 | Licoriphenone | 40 | -5.74 | 37 |
|  | IMPHY008064 | Hemileiocarpin | 2 | -6.97 | 98 |
|  | IMPHY008216 | Licoagrodin | 32 | -8.37 | 19 |
|  | IMPHY008226 | alphaalpha-dihydro-354-trihydroxy-45-diisopentenylstilbene | 45 | -6.00 | 14 |
|  | IMPHY008234 | Glycyrrhisoflavanone | 5 | -7.43 | 95 |
|  | IMPHY008252 | Inositol | 12 | -3.64 | 58 |
|  | IMPHY008508 | Furfuryl acetate | 11 | -4.90 | 24 |
|  | IMPHY008689 | Isovitexin | 28 | -6.49 | 18 |
|  | IMPHY008880 | gamma-Octalactone | 8 | -5.19 | 30 |
|  | IMPHY009035 | Formononetin | 9 | -5.46 | 29 |
|  | IMPHY009114 | Shinflavanone | 9 | -8.11 | 39 |
|  | IMPHY009174 | Semilicoisoflavone B | 5 | -6.62 | 55 |
|  | IMPHY009367 | 1-Pentadecanol | 32 | -3.51 | 17 |
|  | IMPHY009368 | Heptadecane | 10 | -3.70 | 42 |
|  | IMPHY009369 | Nonadecane | 10 | -3.66 | 47 |
|  | IMPHY009375 | Docosane | 11 | -4.79 | 16 |
|  | IMPHY009376 | Glabridin | 7 | -6.90 | 59 |
|  | IMPHY009382 | Heneicosane | 15 | -4.29 | 22 |
|  | IMPHY009386 | Isoglycyrol | 5 | -6.85 | 54 |
|  | IMPHY009389 | Pentadecane | 8 | -3.95 | 45 |
|  | IMPHY009406 | glycyrrhetol | 4 | -8.29 | 58 |
|  | IMPHY009419 | Tridecane | 8 | -4.22 | 32 |
|  | IMPHY009482 | Nonacosane | 14 | -3.21 | 16 |
|  | IMPHY009513 | Tridecanoic acid | 10 | -5.42 | 43 |
|  | IMPHY009624 | Ethyl palmitate | 25 | -5.27 | 16 |
|  | IMPHY009680 | Glyyunnansapogenin B | 5 | -8.69 | 37 |
|  | IMPHY009751 | 1-Heptanol | 14 | -4.18 | 44 |
|  | IMPHY009784 | 2'-Hydroxyacetophenone | 11 | -4.53 | 38 |
|  | IMPHY009823 | gamma-Butyrolactone | 4 | -4.08 | 79 |
|  | IMPHY009824 | gamma-Caprolactone | 2 | -4.86 | 61 |
|  | IMPHY009858 | 1-Undecanol | 23 | -3.54 | 19 |
|  | IMPHY009946 | Benzaldehyde | 3 | -4.50 | 74 |
|  | IMPHY010000 | Dodecane | 6 | -3.93 | 54 |
|  | IMPHY010136 | 18α-hydroxyglycyrrhetic acid | 3 | -9.03 | 94 |
|  | IMPHY010393 | Glucoliquiritin | 56 | -6.29 | 7 |
|  | IMPHY010550 | Naringetol | 13 | -6.03 | 50 |
|  | IMPHY010555 | Glabranin | 16 | -6.27 | 25 |
|  | IMPHY010594 | 3'-Methoxyglabradin | 10 | -6.71 | 53 |
|  | IMPHY010841 | Ethyl linoleate | 26 | -3.86 | 15 |
|  | IMPHY011215 | Tetradecane | 8 | -4.11 | 53 |
|  | IMPHY011265 | Licoagroside A | 58 | -6.01 | 6 |
|  | IMPHY011329 | Liquiritigenin-7,4'-diglucoside | 51 | -6.06 | 7 |
|  | IMPHY011396 | 4-Carvomenthenol | 7 | -4.38 | 55 |
|  | IMPHY011409 | Guaiacol | 11 | -4.26 | 45 |
|  | IMPHY011489 | 7,4'-Dihydroxyflavan | 9 | -6.01 | 35 |
|  | IMPHY011523 | Salicylic acid | 2 | -6.08 | 95 |
|  | IMPHY011557 | 4-Isopropylbenzyl alcohol | 9 | -4.22 | 36 |
|  | IMPHY011599 | Terpinolene | 3 | -4.68 | 59 |
|  | IMPHY011620 | Lutein | 4 | -7.59 | 82 |
|  | IMPHY011707 | beta-Carotene | 3 | -7.64 | 96 |
|  | IMPHY011763 | Anethole | 5 | -4.40 | 61 |
|  | IMPHY011842 | 11-Deoxoglycyrrhetinic acid | 2 | -9.92 | 98 |
|  | IMPHY011884 | Pulegone | 4 | -4.94 | 83 |
|  | IMPHY011887 | Deoxoglabrolide | 4 | -8.09 | 49 |
|  | IMPHY011901 | alpha-thujone | 5 | -4.82 | 74 |
|  | IMPHY011918 | alpha-Glycyrrhizin | 23 | -10.56 | 33 |
|  | IMPHY011933 | Caffeic acid | 10 | -5.57 | 61 |
|  | IMPHY011957 | (+)-delta-Cadinene | 1 | -6.24 | 100 |
|  | IMPHY012003 | Betulinic acid | 4 | -9.29 | 56 |
|  | IMPHY012011 | cis-Caffeic acid | 9 | -6.38 | 37 |
|  | IMPHY012028 | Propionic acid | 1 | -4.78 | 100 |
|  | IMPHY012035 | Anabasine | 2 | -4.80 | 96 |
|  | IMPHY012058 | Linalool | 11 | -4.41 | 29 |
|  | IMPHY012082 | Gamma-nonalactone | 9 | -5.01 | 63 |
|  | IMPHY012083 | gamma-Heptalactone | 7 | -5.36 | 64 |
|  | IMPHY012100 | Nonanoic acid | 9 | -6.00 | 45 |
|  | IMPHY012160 | alpha-Terpineol | 12 | -4.37 | 35 |
|  | IMPHY012226 | 18alpha-Glycyrrhetinic acid | 2 | -10.32 | 98 |
|  | IMPHY012358 | 6-Prenylnaringenin | 13 | -6.03 | 31 |
|  | IMPHY012473 | Lupeol | 1 | -8.35 | 100 |
|  | IMPHY012575 | 1-Methoxyficifolinol | 9 | -7.53 | 78 |
|  | IMPHY012614 | Furfuryl formate | 8 | -4.66 | 44 |
|  | IMPHY012721 | Isoquercitrin | 54 | -4.32 | 9 |
|  | IMPHY012804 | Eicosanyl caffeate | 65 | -3.02 | 8 |
|  | IMPHY012826 | Ambrettolide | 2 | -6.20 | 69 |
|  | IMPHY012891 | Isobavachromene | 10 | -6.55 | 45 |
|  | IMPHY012920 | 2-Furanmethanol | 5 | -4.63 | 80 |
|  | IMPHY012954 | Licuroside | 65 | -5.45 | 46 |
|  | IMPHY013522 | Triterpenoids | 6 | -8.57 | 32 |
|  | IMPHY013718 | Kanzonol R | 20 | -6.78 | 44 |
|  | IMPHY013759 | Hydroxyacetone | 8 | -3.42 | 65 |
|  | IMPHY013836 | Fenchone | 4 | -5.14 | 97 |
|  | IMPHY014396 | Quercetin-3-glucoside | 53 | -4.65 | 15 |
|  | IMPHY014675 | 1-Methyl-3-nitro-1-nitrosoguanidine | 8 | -5.68 | 73 |
|  | IMPHY014824 | Astragalin | 49 | -4.76 | 10 |
|  | IMPHY014836 | beta-Sitosterol | 7 | -8.64 | 32 |
|  | IMPHY014842 | Stigmasterol | 11 | -8.43 | 29 |
|  | IMPHY014916 | D-Fructose | 14 | -5.06 | 64 |
|  | IMPHY014916 | D-Fructose | 14 | -5.06 | 64 |
|  | IMPHY014923 | Geraniol | 10 | -4.57 | 46 |
|  | IMPHY014926 | Enoxolone | 6 | -9.88 | 57 |
|  | IMPHY014927 | Glycyrrhizic acid | 28 | -8.25 | 17 |
|  | IMPHY014989 | trans-Linalool oxide | 5 | -4.59 | 72 |
|  | IMPHY014998 | beta-Maltose | 46 | -3.53 | 8 |
|  | IMPHY015004 | Menthone | 4 | -4.71 | 79 |
|  | IMPHY015047 | Rutin | 80 | -4.71 | 7 |
|  | IMPHY015054 | Quercitrin | 42 | -5.78 | 16 |
|  | IMPHY015465 | Abssinone II | 23 | -6.95 | 30 |
|  | IMPHY015534 | Butyric anhydride | 8 | -5.29 | 75 |
|  | IMPHY015688 | 2,4-Difurfurylfuran | 11 | -7.13 | 26 |
|  | IMPHY015689 | Furfuryl butyrate | 13 | -5.30 | 29 |
|  | IMPHY015690 | Difurfuryl ether | 7 | -5.79 | 37 |
|  | IMPHY015692 | Furfuryl propionate | 11 | -5.25 | 34 |
|  | IMPHY015702 | Glabric acid | 3 | -9.67 | 85 |
|  | IMPHY015703 | Glyinflanin H | 8 | -6.04 | 37 |
|  | IMPHY015707 | Isoglycycoumarin | 6 | -6.67 | 61 |
|  | IMPHY015709 | 28-Hydroxyglycyrrhetic acid | 5 | -9.21 | 83 |
|  | IMPHY015710 | Glyinflanin G | 13 | -7.74 | 29 |
|  | IMPHY015715 | 1,2-Heptanediol | 15 | -3.66 | 28 |
|  | IMPHY015779 | Kanzonol B | 5 | -7.18 | 85 |
|  | IMPHY015780 | Kanzonol T | 13 | -6.42 | 38 |
|  | IMPHY015781 | Kanzonol U | 6 | -6.40 | 56 |
|  | IMPHY015782 | Kanzonol V | 19 | -6.99 | 28 |
|  | IMPHY015783 | Kanzonol W | 7 | -6.68 | 77 |
|  | IMPHY015784 | Kanzonol X | 19 | -7.15 | 33 |
|  | IMPHY015792 | Licoagrocarpin | 9 | -6.18 | 36 |
|  | IMPHY015793 | Licoflavanone | 24 | -6.43 | 23 |
|  | IMPHY015794 | Licoisoflavanone | 11 | -7.04 | 35 |
|  | IMPHY015849 | Isomucronulatol | 9 | -5.33 | 40 |
|  | IMPHY015890 | (S)-8-Prenylphaseollinisoflavan | 19 | -7.16 | 22 |
|  | IMPHY015890 | (S)-8-Prenylphaseollinisoflavan | 19 | -7.16 | 22 |
|  | IMPHY015893 | 2-Phenylbutanoate | 1 | -6.33 | 100 |
|  | IMPHY015919 | 2-Ethyl-6-methylpyrazine | 4 | -4.68 | 57 |
|  | IMPHY015920 | Pyrazole | 4 | -3.17 | 62 |
|  | IMPHY015988 | Xambioona | 4 | -7.41 | 77 |
|  | IMPHY016286 | Methacrylonitrile | 3 | -3.72 | 81 |
|  | IMPHY016827 | Dehydroepiandrosterone | 4 | -7.21 | 77 |
|  | IMPHY016835 | Pyrazine, 1,4-dioxide | 2 | -4.48 | 96 |
|  | IMPHY017165 | 4-Aminopyridine | 9 | -3.68 | 41 |
|  | IMPHY017205 | 3,5-Dimethylstyrene | 3 | -5.46 | 73 |
|  | IMPHY017284 | 4-(2-Aminopropyl)phenol | 7 | -4.58 | 36 |
|  | IMPHY017676 | 2'-Methoxyacetophenone | 6 | -4.61 | 70 |

**Table S3:** **52 phytoconstituents of *Tinospora cordifolia***

| **Sl. No.** | **IMMPAT ID** | **Phytochemical Name** | **No. of conformation** | **Lowest free energy of binding in largest cluster** | **Number of conformations in largest cluster** |
| --- | --- | --- | --- | --- | --- |
|  | IMPHY000060 | Myristic acid | 9 | -5.35 | 29 |
|  | IMPHY000165 | Tetracosanoic acid | 27 | -5.94 | 23 |
|  | IMPHY000245 | Cordioside | 39 | -6.08 | 16 |
|  | IMPHY000511 | Cardiofolioside B | 70 | -2.67 | 8 |
|  | IMPHY000768 | Xanosporic acid | 23 | -5.75 | 21 |
|  | IMPHY000801 | Tinosinen | 62 | -3.64 | 5 |
|  | IMPHY000801 | Tinosinen | 9 | -5.58 | 44 |
|  | IMPHY001207 | Kokusaginine | 6 | -7.00 | 63 |
|  | IMPHY001347 | Cordifolide A | 63 | -5.56 | 9 |
|  | IMPHY001506 | Tinosponone | 5 | -6.69 | 50 |
|  | IMPHY001896 | Heptacosane | 13 | -2.48 | 32 |
|  | IMPHY001971 | 1-Penten-3-OL | 6 | -3.55 | 41 |
|  | IMPHY002667 | Pentadecanoic acid | 14 | -5.52 | 30 |
|  | IMPHY002960 | Tinosporaside | 23 | -6.96 | 18 |
|  | IMPHY002976 | Tinosporinone | 18 | -6.93 | 27 |
|  | IMPHY003271 | Malabarolide | 7 | -5.58 | 62 |
|  | IMPHY003499 | Pyrrolidine | 6 | -3.61 | 60 |
|  | IMPHY003525 | Nonanal | 17 | -3.69 | 25 |
|  | IMPHY003844 | 2-Penten-1-OL | 7 | -3.76 | 36 |
|  | IMPHY004388 | Kaempferol | 18 | -5.25 | 26 |
|  | IMPHY004977 | Syringin | 47 | -3.80 | 7 |
|  | IMPHY005103 | Tembetarine | 21 | -5.43 | 22 |
|  | IMPHY005330 | Palmatine | 2 | -6.13 | 92 |
|  | IMPHY005665 | Berberine | 7 | -6.15 | 76 |
|  | IMPHY006165 | 1-Heptacosanol | 51 | -5.14 | 5 |
|  | IMPHY006959 | Hydroquinone | 6 | -4.25 | 56 |
|  | IMPHY006972 | 1-Octacosanol | 53 | -2.92 | 7 |
|  | IMPHY007171 | 1-Hexanol | 9 | -3.91 | 22 |
|  | IMPHY007190 | Jatrorrhizine | 5 | -6.03 | 59 |
|  | IMPHY007327 | Palmitic acid | 17 | -5.10 | 30 |
|  | IMPHY008571 | Menispermacide | 5 | -6.90 | 38 |
|  | IMPHY008905 | Escholine(magnoflorine) | 4 | -5.78 | 90 |
|  | IMPHY009359 | Hexacosane | 13 | -4.40 | 13 |
|  | IMPHY009377 | Pentacosane | 15 | -4.24 | 10 |
|  | IMPHY009481 | Octacosane | 17 | -4.07 | 20 |
|  | IMPHY011562 | 2-Hexenal | 3 | -4.31 | 52 |
|  | IMPHY011588 | cis-3-Hexen-1-ol | 11 | -4.06 | 34 |
|  | IMPHY011650 | Chasmanthin | 8 | -6.75 | 12 |
|  | IMPHY011694 | 2-Hexen-1-OL | 9 | -4.15 | 34 |
|  | IMPHY011716 | Palmarin | 4 | -7.71 | 55 |
|  | IMPHY011716 | Palmarin | 4 | -7.71 | 55 |
|  | IMPHY011813 | columbin | 6 | -7.40 | 48 |
|  | IMPHY011836 | Tinosporin | 4 | -7.99 | 52 |
|  | IMPHY011861 | 15-Nonacosanone | 28 | -3.67 | 11 |
|  | IMPHY012029 | Sterol | 2 | -6.74 | 98 |
|  | IMPHY012058 | Linalool | 12 | -5.18 | 20 |
|  | IMPHY012712 | Phytol | 34 | -4.56 | 16 |
|  | IMPHY012895 | 2,4-Pentadienal | 3 | -3.92 | 89 |
|  | IMPHY014396 | Quercetin-3-glucoside | 45 | -4.79 | 21 |
|  | IMPHY014824 | Astragalin | 49 | -5.50 | 8 |
|  | IMPHY014824 | Astragalin | 49 | -5.50 | 8 |
|  | IMPHY014836 | beta-Sitosterol | 9 | -8.56 | 54 |

**Table S4:** **18 phytoconstituents of *Convolvulus pleuricaulis***

| **Sl. No.** | **IMMPAT ID** | **Phytochemical Name** | **No. of conformation** | **Lowest free energy of binding in largest cluster** | **Number of conformations in largest cluster** |
| --- | --- | --- | --- | --- | --- |
|  | IMPHY006558 | 1-Triacontanol | 54 | -2.79 | 6 |
|  | IMPHY006972 | 1-Octacosanol | 53 | -3.62 | 6 |
|  | IMPHY007273 | 1-Hexacosanol | 45 | -3.06 | 6 |
|  | IMPHY009413 | Triacontane | 18 | -3.46 | 19 |
|  | IMPHY009485 | Pentatriacontane | 19 | -2.01 | 21 |
|  | IMPHY010556 | Scopoline | 5 | -3.67 | 50 |
|  | IMPHY011541 | Scopoletin | 11 | -5.91 | 16 |
|  | IMPHY011933 | Caffeic acid | 10 | -5.56 | 60 |
|  | IMPHY012478 | Convolvine | 5 | -6.41 | 69 |
|  | IMPHY012507 | Convolamine | 9 | -5.90 | 61 |
|  | IMPHY012560 | Convoline | 20 | -5.80 | 21 |
|  | IMPHY012704 | Convolvidine | 67 | -5.66 | 5 |
|  | IMPHY013995 | convolidine | 13 | -6.45 | 36 |
|  | IMPHY014035 | Subhirsine | 39 | -6.30 | 11 |
|  | IMPHY006558 | 1-Triacontanol | 54 | -2.79 | 6 |
|  | IMPHY007273 | 1-Hexacosanol | 45 | -3.06 | 6 |
|  | IMPHY012560 | Convoline | 20 | -5.80 | 21 |
|  | IMPHY014836 | beta-Sitosterol | 10 | -8.85 | 41 |
